# Ecomorphological relationships and invasion history of non‐native terrestrial bird species on O‘ahu, Hawai‘i, suggest ecological fitting during novel community assembly

**DOI:** 10.1002/ece3.6843

**Published:** 2020-10-03

**Authors:** Jason M. Gleditsch, Jinelle H. Sperry

**Affiliations:** ^1^ Department of Natural Resources and Environmental Sciences University of Illinois at Urbana‐Champaign Urbana IL USA; ^2^ Engineer Research and Development Center U.S. Army Corps of Engineers Champaign IL USA

**Keywords:** ecomorphology, foraging ecology, Hawaii, introduced species, novel communities, Oahu

## Abstract

The widespread introduction of species has created novel communities in many areas of the world. Since introduced species tend to have generalized ecologies and often lack shared evolutionary history with other species in their communities, it would be expected that the relationship between form and function (i.e., ecomorphology) may change in novel communities. We tested this expectation in a subset of the novel bird community on O‘ahu, Hawai‘i. By relating foraging behavior observations to morphology obtained from live birds at four sites across the island, we found many relationships between species’ morphology and foraging ecology that mirrored relationships found in the literature for native‐dominated bird communities. Both movement and certain foraging behaviors were related to a species’ tarsus‐to‐wing ratio. Further, bill morphology was related to gleaning, frugivory, and flycatching behaviors. The commonness of significant ecomorphological relationships suggests that, within O‘ahu's novel bird community, form is strongly related to function. We hypothesize that ecological fitting likely played a major role in the assembly of this novel community conserving the relationships between form and function found in many other bird communities. To further support this hypothesis, we used niche data from EltonTraits 1.0 to determine whether the establishment of bird species introduced to O‘ahu was related to the distinctiveness of their ecological niche from the incumbent community. Introduced species were more likely to establish on O‘ahu if their diets were less similar to the bird species already present on the island. Our results support the idea that ecological fitting is an important mechanism in shaping ecological communities, especially in the Anthropocene, thereby influencing novel community assembly and functioning.

## Graphical abstract

We related foraging behavior observations to morphology obtained from live birds at four sites across Oahu, Hawaii, and found many relationships between species' morphology and foraging ecology that mirrored relationships found in native‐dominated bird communities. This along with our result that introduced species were more likely to establish on O‘ahu if their diets were less similar to the bird species already present on the island suggest that ecological fitting is the main driver in the assembly of novel ecological communities.

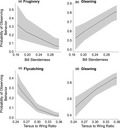

## 1 INTRODUCTION

The spread and introduction of species has created a novel mix of interacting species in most ecosystems worldwide (Vitousek et al., 1997). While the effects of introduced species on native communities and species have been a focus of conservation science over the past half century (Pyšek et al., 2012), the functioning and stability of these novel communities has only started to be explored. Novel ecosystems, by definition, lack shared evolutionary history, and therefore, comprise a collection of species that have not evolved to be in a certain ecosystem and/or ecological community (Hobbs et al., 2006; Lugo et al., 2012). As a result, the ecology and evolution of non‐native species in novel communities can be altered from their native ranges (Stiels et al., 2015), influencing the ability of these species to fulfill their ecological roles. When assessing the functional roles species play in an ecosystem, morphology is often used as a proxy (Ricklefs, 2012). The relationship between a species’ function and their morphology—termed ecomorphology—is based on the idea that a species’ morphology has evolved to fit their specific environment and ecological niche (Ricklefs & Miles, 1994). Within novel ecosystems, species lack this evolutionary history and, therefore, may have a morphology that does not ideally match their ecological characteristics leading to weak or nonexistent ecomorphological relationships.

Ecomorphological relationships have been shown in many native‐dominated systems and taxonomic clades. In some bird clades, researchers have found exceptionally strong relationships between foraging ecology and morphology (Leisler & Winkler, 1985; Miles & Ricklefs, 1984; Miles et al., 1987), leading to morphology accurately predicting ecological functions across the Avian phylogeny (Pigot et al. 2020). Bill morphology, for instance, has a well‐established link to what and how birds eat (Felice et al., 2019; Schluter & Grant, 1984; Tokita et al., 2017). While some species may overcome some morphological constraints with behavioral adaptation (Diogo, 2017), a bird's bill morphology often determines what it can and cannot eat efficiently (Jordano, 2000; Leisler & Winkler, 1985). Some general trends have been suggested such as shorter, thinner bills being characteristic of specialization for foliage gleaning while deeper more robust bills are characteristic of a more generalized diet (Clegg & Owens, 2002; Leisler & Winkler, 1985; Moermond & Denslow, 1985). Similarly, studies have shown shorter wings and longer tarsi are better adapted for movement through the dense vegetation of the forest understory with the reverse being better adapted for longer flights during foraging (Savile, 1957). However, mismatches in ecomorphological relationships can develop when a shift in a species’ ecology or environment occurs (Diogo, 2017), such as those experienced following introductions of non‐native species into insular systems.

It is thought that when species are introduced to islands, they undergo a niche expansion, due to enemy release, resulting in a more generalized ecology (Blondel et al., 1988; MacArthur et al., 1972). Indeed, the Indian mongoose has exhibited a broadening of its dietary niche on islands where it has been introduced due to a reduction in competition with other mesopredators (Simberloff et al., 2000). Further, species present in highly dynamic ecosystems, such as many oceanic islands following human discovery and associated anthropogenic disturbance (e.g., Hawai‘i, Athens et al., 2002), tend to have more generalized ecologies (Clavel et al., 2011; Kassen, 2002). The generalization of the species’ ecology within a community can thus erode the ecomorphological relationships by allowing individuals within a population to exhibit different foraging behaviors (Bolnick et al., 2007). Additionally, the successful establishment of introduced species in novel systems is often linked to behavioral changes during the introduction process (Holway & Suarez, 1999; Wright et al., 2010), which may further decouple the relationship between a species’ morphology and its ecology. Therefore, it may be expected that ecomorphological relationships within novel communities on highly disturbed islands may be lacking or different from those in native‐dominated systems.

On the other hand, as novel communities assemble on islands, a non‐native species may only become established if it possesses a morphological and ecological phenotype that allows it to perform well in the available niche space (i.e., ecological fitting; Janzen, 1985), a process thought to be common in the integration of non‐native species into communities (Sax et al., 2007). Ecological fitting occurs through complex environmental and biotic interactions, such as competition with other species within the incumbent community, and therefore, species may only become established if they have ecologies that are distinct enough to exploit the available niche space in the presence of a novel assemblage of interacting species (Agosta & Klemens, 2008). This process would, ultimately, lead to a complex community that retains many ecomorphological relationships similar to communities that have extensive evolutionary history (i.e., native‐dominated).

Insular systems are often characterized as being species depauperate (Rosenzweig, 1995), which can be exasperated by the extinctions of native species due to overhunting, habitat loss, and introductions of predators and parasites (Vitousek et al., 1987). The Hawaiian Archipelago, and the island of O‘ahu in particular, presents an extreme example of species extinction and invasion in an insular system (Vitousek et al., 1987). Currently, the passerine community in the forests of O‘ahu includes only three native species (‘apapane—*Himatione sanguinea*; O‘ahu amakihi—*Chlorodrepanis flavus*; O‘ahu ‘elapaio—*Chasiempis ibidis*; Pyle & Pyle, 2017), one of which, the O‘ahu ‘elapaio, is endangered (estimated population size of approximately 1,261 individuals; BirdLife International, 2016), making the community almost entirely novel. The great loss of species on O‘ahu (83% of the 30 native‐resident land bird species known historically and from subfossils; Walther, 2016) likely resulted in unoccupied niche space for non‐native species to fill and may have led to a loss or reduction in performance of certain functional roles within communities (Dehling et al., 2016). For instance, all of the native frugivores (i.e., animals that eat fruit and disperse seeds) have gone extinct or have been extirpated from O‘ahu (Heinen et al., 2018) and were predominantly replaced by four non‐native passerine species (Japanese white‐eye—*Zosterops japonicus*, red‐billed leiothrix—*Leiothrix lutea*, red‐vented bulbul—*Pycnonotus cafer*, and red‐whiskered bulbul—*Pycnonotus jocosus*; Vizentin‐Bugoni et al., 2019). However, the non‐native species in Hawai'i are only partially fulfilling the functional roles of the now extinct native frugivores (Aslan et al., 2014; Chimera & Drake, 2010; Vizentin‐Bugoni et al., 2019), in large part due to morphological mismatch between seed size and gape width (Pejchar, 2015 and see Case & Tarwater, 2020 for a detailed comparison of native and non‐native morphologies). Understanding the ecomorphological relationships of these non‐native species is critical for understanding and predicting plant–animal interactions and ecosystem functioning in this novel system. However, the necessary behavioral data for these introduced species are not available from their native range, and without these data, direct comparisons to their ecomorphological relationships in their native range are not possible.

Here, we investigated the morphology and behavior of the four primary frugivores and one primarily insectivorous bird species on the Hawaiian island of O‘ahu. We tested predictions derived from ecomorphological relationships that have been established in native‐dominated systems (Table 1) to determine whether non‐native species on O‘ahu had similar (suggesting ecological fitting and/or adaptation) or differing (suggesting niche expansion and/or shifts) ecomorphological relationships, in relation to direction and existence, to native‐dominated bird communities. The use of literature‐derived relationships is appropriate since these relationships have been shown in many systems and across the avian phylogeny (Pigot et al., 2020). Further, using historic data on the bird introduction history of O‘ahu, we investigated the potential role of ecological fitting in the creation of the novel bird community now present on the island. We then discuss how the rapid evolution of the frugivore species, as observed in Gleditsch and Sperry (2019), may influence these relationships and community functioning.

**TABLE 1:** The predictions based on the literature used to build the models between morphology and ecology

Prediction	Behavior	Tarsus to wing	Horiz. Bill aspect	Bill slenderness	Mass	Num. of species
1	Foraging Height^1−10^	Neg			Neg	5
2	Gleaning^11^	Pos	Pos^a^	Pos		4
3	Flycatching^11,12^	Neg	Neg			4
4	Frugivory^13,14^			Neg	Pos	4
5	Hanging^15^	Neg			Neg	5
6	Hopping^15^	Pos				5
7	In Periphery^11,15,16^	Neg			Neg	5
8	On Ground^15^	Pos			Pos	5

Note

The blanks represent relationships that were not described in the literature used for determining our predictions. Also shown are the number of species used to test each prediction. See text for how the morphological characteristics were calculated.

^1^Osterhaus (1962), ^2^Grant (1965), ^3^Grant (1966), ^4^Fretwell (1969), ^5^Gaston (1974), ^6^Cody and Mooney (1978), ^7^Saether (1983), ^8^Miles and Ricklefs (1984), ^9^Landmann and Winding (1993), ^10^Forstmeier and Keßler (2001), ^11^Leisler and Winkler (1985), ^12^Price (1991), ^13^Moermond and Denslow (1985), ^14^Snow and Snow (1988), ^15^Zeffer et al. (2003), ^16^Savile (1957).

Abbreviations: Neg, negative relationship; Pos, positive relationship.

^a^ Only bill slenderness was used in the model for prediction 2 because bill horizontal aspect ratio and bill slenderness are correlated.

## 2 MATERIALS AND METHODS

### 2.1 Study system

Our research took place on the Hawaiian Island of O‘ahu where we focused our observations on five non‐native species, four of which are the primary seed dispersers, a vital ecosystem function (Fleming & Kress, 2013), in the forests of O‘ahu (Vizentin‐Bugoni et al., 2019). The four frugivores we focused on were the Japanese white‐eye (hereafter JAWE), red‐billed leiothrix (hereafter RBLE), red‐vented bulbul (hereafter RVBU), and the red‐whiskered bulbul (hereafter RWBU) and are among the most abundant bird species in the forests of O‘ahu (Gleditsch, 2019). The fifth species, white‐rumped shama (*Copsychus malabaricus*; hereafter WRSH), is primarily insectivorous and acts as an outlier to suggest whether the relationships we observed can be extended outside of the frugivore guild. It should be noted that all five species are primarily invertivores or omnivorous and not primarily frugivorous with fruit only making up approximately 10%–30% of their diets (Wilman et al., 2014) with WRSH at the lower end of that range (19 of 299 fecal samples had seeds; Vizentin‐Bugoni et al., 2019). However, WRSH were rarely observed foraging so they were removed from the analyses of the relationships between morphology and foraging behaviors (i.e., gleaning, frugivory, and flycatching) and were only used in the analyses of movement behaviors (i.e., foraging height, hopping, hanging, on the ground, and in the periphery of plant). The five focal species used in this study made up 63%–80% of the bird communities of 18 – 20 species at point counts in each of the study sites, with JAWE, RBLE, RVBU, and RWBU almost always the top four species (60%–76% of the community) by abundance (Gleditsch, 2019).

### 2.2 Foraging observations

To determine the foraging behavior of these species, we conducted observations at four sites from January through July in 2016 and 2017. Since El Niño events can influence bird populations in Hawai‘i (Lindsey et al., 1997), it is important to note that 2016 was the decaying period of a particularly strong El Niño event (Santoso et al., 2017) resulting in the potential for variable abiotic conditions between the two study years. The four sites were Ekahanui Valley (21°26ʹ36.98″N, 158°04ʹ52.11″W; hereafter EKA), Pahole Natural Area Reserve (21°31ʹ56.24″N, 158°10ʹ42.97″W; hereafter PAH), Waimea Valley (21°37ʹ49.97″N, 158°01ʹ49.59″W; hereafter WAI), and Moanalua Valley (21°22ʹ37.77″N, 157°52ʹ16.62″W; hereafter MOA). The sites ranged in elevation (94–667 m above sea level) and mean annual rainfall (1107–1884 mm). In addition, the sites varied in their plant and bird communities (Vizentin‐Bugoni et al., 2019). The sites were visited every other week with 13–15 days between visits. Visits started approximately at sunrise and concluded before 1300. Depending on weather, four observations were conducted during each visit to each site at four randomly selected points from 9–13 (proportional to size of site) previously established points at least 150 m apart. Observations were not conducted in heavy rain due to altered behaviors and lack of foraging activity. After the same four points were visited twice, a new set of four points was randomly chosen (with replacement).

At each point, the observation period lasted one hour. During the one‐hour observation, all observations of the five focal species were recorded including the bird's maximum and minimum height in the tree (visually estimated to the nearest 1.5 m—i.e., 5 ft), location (interior or periphery) in the tree, foraging behaviors (gleaning, flycatching, fruit consumption, and nectar consumption), foraging posture (upright or hanging), mode of movement (hop, walk, flight), and any other behaviors. In addition, the plant species the bird was interacting with was recorded using an intensity of interaction score (0 = no interaction, 1 = quickly move through, 2 = perching/singing, 3 = foraging in the plant on arthropods, 4 = consumption of plant produced resource—i.e., fruit or nectar). If the bird was in a flock, the flock size was noted and the height range for the flock was recorded. Competitive interactions were also recorded. To control for the variation in the canopy heights, the heights in the forest the birds were observed were transformed to relative heights by dividing the height the bird was observed by the average canopy height for each point (as measured in Gleditsch, 2019). The foraging behaviors and the modes of movement were recorded as presence–absence variables. By doing this, each observation could include more than one observed behavior or movement type. To be able to capture the most behaviors, the observations were dictated into a voice recorder and then later transcribed.

### 2.3 Morphological data collection

To determine the relationships between the species’ morphology and ecology, we used five morphological characters, including wing length, tarsus length, culmen length, and bill width and depth at the nares, as taken in Gleditsch and Sperry (2019) for each species at each site. Mist netting was conducted at each site from November 2014 through December 2017, and the five morphological measurements were taken on as many individuals as possible focusing on the five focal species. Mass was also collected for each bird species by weighing the birds using spring scales (PESOLA, Switzerland) in a bag and subtracting the mass of the bag. Morphological measurements of juvenile birds were not used in any analysis.

From the morphological data, we calculated morphological ratios (i.e., tarsus‐to‐wing ratio; and bill aspect ratios). The bill aspect ratios were calculated in two different ways: horizontal aspect ratio calculated by dividing the culmen length by the width of the bill at the proximal end of the nares, and the three‐dimensional aspect ratio calculated by dividing the culmen length by the cross‐sectional area of the bill at the proximal end of the nares (hereafter bill slenderness). For bill slenderness, a lower value means that the bill is more robust (i.e., shorter and fatter) and a higher value the bill is slenderer (i.e., longer and thinner). Due to a low rate of resighted birds (birds were color banded for another study), we averaged the morphological ratios for each species at each site.

### 2.4 O‘ahu introduction history data

To determine whether ecological fitting may have been an important process during community assembly, we compared resource use distributions (in this case diet and foraging strata) between birds introduced to O‘ahu (successfully and unsuccessfully) and those already present at the time of introduction. We obtained diet resource and foraging strata use distributions for each terrestrial bird species that was reported by Pyle and Pyle (2017) to have been introduced to O‘ahu (126 species—40 of which are considered established). Additionally, we obtained the same information for every native species documented in Pyle and Pyle (2017) for which the information was available (12 species). Even though there are more known extinctions of native bird species on O‘ahu (Walther, 2016), many of them are only known from the fossil record, and therefore, there is little information about their ecology. The resource use information for all species was obtained from EltonTraits 1.0 (Wilman et al., 2014). To obtain this information, Wilman and colleagues mined various texts (e.g., articles, accounts, and guides) and scored each resource type according to the language used and order of description in the text. For each species, they gave a certainty score for the distribution of resources used in the species’ diet. This ranged from high certainty (A), reasonably certain (B), unclear quality (C), and species lacking information so values represent typical values from genus (D1 or D2). For the 134 species that we were able to find data for, 115 were in certainty category A, 7 were in B, 3 were in C, and 9 were in D1. We could not find diet information for 4 native species that were considered extinct either before or shortly after European discovery of Hawai‘i. In addition to the diet resource distributions, EltonTraits also has foraging strata use distributions obtained in a similar way to diet, which we also used to determine whether differences in foraging strata used influenced the probability of establishment. In order to determine the incumbent community at the time a species was introduced, we needed each species’ introduction and, if applicable, extirpation dates. For a species’ introduction date, we used dates provided by Pyle and Pyle (2017) and used the first reported date if an introduction date was not explicitly provided. The extirpation dates were considered to be the last year the species was reported. When dates were not explicitly stated in Pyle and Pyle (2017), we used other sources (Moulton, 1985, 1993; Moulton & Pimm, 1983; Simberloff & Boecklen, 1991) including eBird (Sullivan et al., 2009) for the last reported dates. See Appendices S1–S3 for the EltonTraits and introduction history data.

### 2.5 Statistical analysis

To determine whether the differences in ecological niche of the non‐native bird community on O‘ahu are related to the difference in morphology, we first ran a principal component analysis (PCA) on the raw morphological data with the individual bird as the experimental unit. From this analysis, we calculated the Euclidean distance (hereafter *D*_E_) between each of the species’ centroids at each site to determine the similarity of their morphologies. The similarity between the species’ foraging niche at each site was determined by calculating the proportional similarity index (hereafter PS; Feinsinger et al., 1981) between the relative frequency distributions of the various observed foraging behaviors (i.e., gleaning, flycatching, nectar feeding, frugivory). The proportional similarity index ranges from the minimum relative frequency a behavior is observed, meaning the distributions are the most different, to one, which corresponds to no difference in the distributions. The PS is calculated using the following equation:

$$\mathrm{PS}=\sum_{i}\min\left(p_{i},q_{i}\right)$$

where $p_{i}$ is the proportion of resource (or behavior) *i* used by species *p* and $q_{i}$ is the proportion of resource (or behavior) *i* used by species *q*.

We used the *D*_E_ and PS to determine whether there was a relationship between the species’ similarity in foraging niche and the similarity in their morphologies by running a quasibinomial generalized linear model (without WRSH) with PS as the dependent variable and the *D*_E_ as the independent variable. We checked to see whether including site as a random effect was necessary and found that including site as a random variable was not necessary. In order to determine whether the relationship between morphology and ecology extends beyond our focal species to the rest of the bird community, we repeated this analysis at the species level with EltonTraits 1.0 data for all the species for which we collected morphology data during mist netting (17 species), which included two native species (see Appendix S4 for the species included in this model).

Generalized linear mixed models were performed with site and species as random variables and a binomial error distribution to determine the ecomorphological relationships within the focal bird community. The dependent variable in each model was the specific behaviors, and the independent variable was the site‐averaged morphological ratios. The morphological variables for each model were selected using information obtained through literature review of avian ecomorphological relationships (Table 1). Even though certain behaviors can be predicted to have relationships to multiple bill characteristics (see Table 1), the ratios that we calculated were all highly correlated with each other, and therefore, we only chose one depending on which one had more literature linking it to the specific behavior. To determine the relationship between the height the bird was observed and their morphology, we performed a normal linear mixed model with site and species as random variables and relative height as the dependent variable. For the relationship between height and morphology, we split the height into maximum observed height for an observation and minimum observed height for an observation, resulting in two different height models. We did this to account for the variation in the range of heights species were observed (e.g., JAWE was often observed from one meter to above the average canopy in an emergent tree while RVBU was almost always near or above the canopy).

To determine the potential importance of ecological fitting, we calculated PS indices for diet resource distributions and foraging strata (as an index for niche overlap) for each pair of the 134 species listed by Pyle and Pyle (2017) and then averaged the indices for each species that was present when a particular species was reported to be first introduced to the island (i.e., incumbent community). In order to determine the incumbent community for each introduced species, we selected each species that was introduced (and native species) before or during the year the species was introduced and has not been extirpated from the island. To relate the average similarity in resource use distributions between introduced species and incumbent communities, we used a binomial generalized mixed models with the average foraging strata PS (hereafter PS_for_), average diet PS (hereafter PS_diet_), number of years in which the species was introduced (as a proxy for propagule pressure), and the incumbent community's species richness as predictors. The response variable was whether or not the species successfully established as determined by Pyle and Pyle (2017), and we included a random factor of the species' taxonomic family. Because multiple game bird species (20 species belonging to three families: Phasianidae, Numididae, and Odontophoridae) were introduced to the island for hunting purposes, their success may have been a function of hunting pressure rather than their ecological, or morphological, characteristics. Therefore, we repeated this analysis excluding the game birds. Additionally, species where only one individual was reported to have been introduced were also excluded.

## 3 RESULTS

We conducted a total of 121 hr of observations which resulted in 268 foraging observations, 576 observations of movement, and 695 observations of the birds’ height in the forest. Of these observations, 232 of them were of groups (range 2–10 birds) and 510 were of a single bird. Thirty plant species were observed being foraged in or on, with four plant species native to O‘ahu. Japanese white‐eyes foraged in the most plant species (*n* = 23) followed by RBLE (*n* = 18), RWBU (*n* = 17), and RVBU (*n* = 13). White‐rumped shamas were only observed foraging three times and only in *Hibiscus tiliaceus*. The plant species most often observed being foraged in or on were *Psidium cattleyanum* (*n* = 27) and *Schinus terebinthifolius* (*n* = 25) both of which are very common non‐native plants. Of the native plant species included in this study, *Metrosideros polymorpha* (*n* = 18) and *Acacia koa* (*n* = 8) were most often foraged in.

**FIGURE 1:**
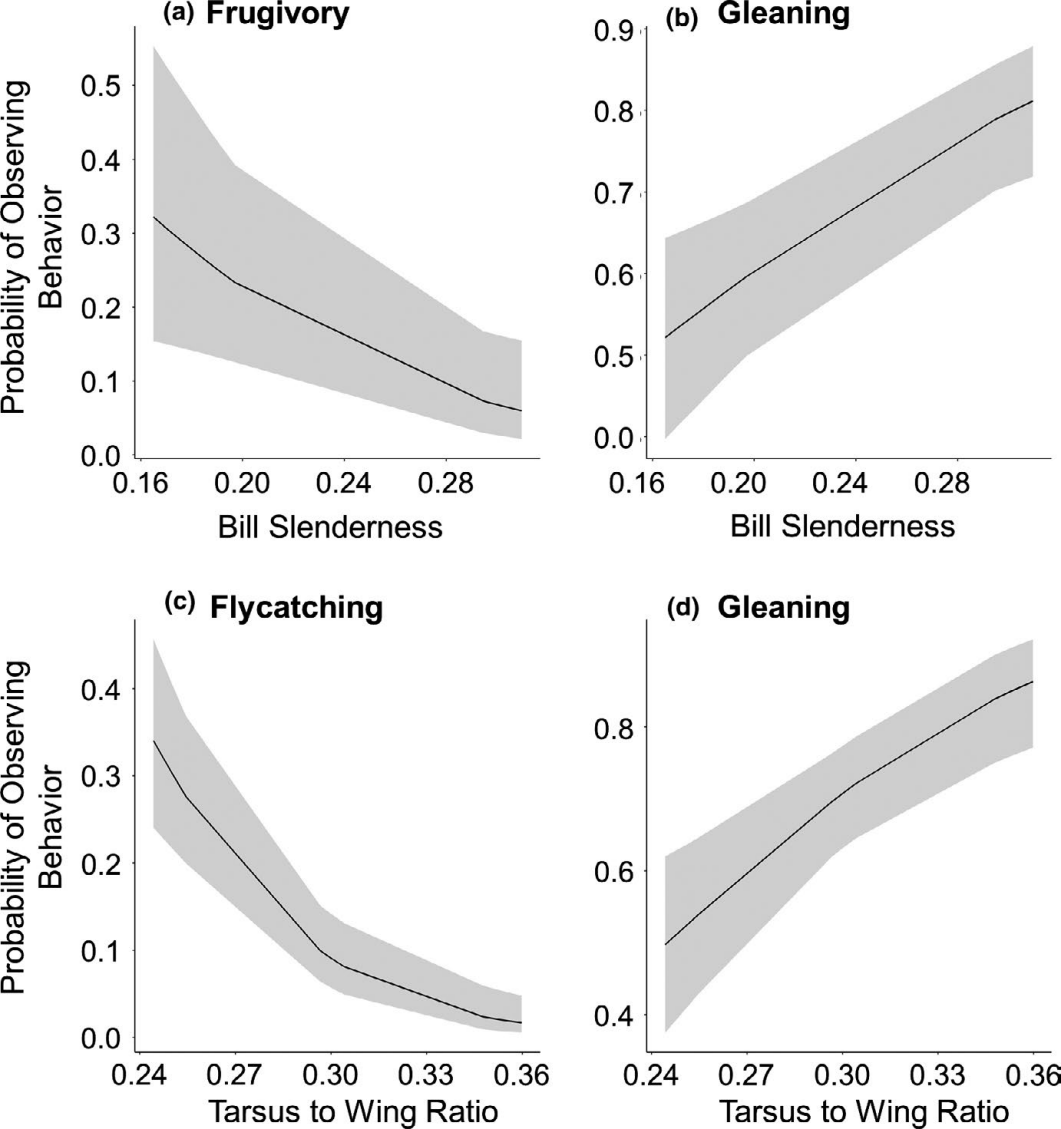
Predicted relationships from generalized linear mixed models between bill slenderness and (a) the probability of observing frugivory and (b) the probability of observing gleaning behaviors. Bill slenderness was calculated by dividing bill length by the cross‐sectional area at nares, and therefore, smaller values represent more robust bills and larger values represent slenderer bills. Also shown are the predicted relationships between the tarsus‐to‐wing ratio and (c) the probability of observing flycatching behaviors and (d) the probability of observing gleaning behaviors. The gray ribbons represent 95% confidence intervals

We found a significant relationship between morphology and foraging niche. Within our focal species, species with more similar morphology (smaller *D*_E_) had a more similar foraging ecology (higher PS) (estimate = −0.221; *SE* = 0.103; *t* = −2.1.34; *df* = 16; *p*‐value = .049). When we expanded our analysis to include species outside of the frugivore community, we found a similar relationship with species that had more similar diets (higher PS_diet_) also having more similar morphology (estimate = −0.130; *SE* = 0.059 *t* = −2.198; *df* = 134; *p*‐value = .030). Additionally, we found several significant relationships between morphology and foraging behavior (Table 2). The probability of observing gleaning behaviors during foraging was significantly related to bill slenderness and the tarsus‐to‐wing ratio with species that had slenderer bills and longer tarsi in relation to their wings exhibiting more gleaning behaviors (Table 2; Figure 1a,d). Conversely, species with wider bills and longer wings in relation to their tarsi were more likely to exhibit flycatching behaviors (Table 2; Figure 1c). The species that had more robust bills were observed eating fruit more often than birds with slenderer bills (Table 2; Figure 1b). However, observing frugivory was not related to a species’ mass (Table 2).

**TABLE 2:** Results from the generalized linear mixed models of three observed foraging behaviors and morphology

Prediction	Response variable	Morphology	Estimate	*SE*	*t*‐Value	*p*‐Value
2	Gleaning	**Bill Slenderness**	**9.496**	**2.711**	**3.503**	**.0005**
	(4 species)	**Tarsus:Wing**	**16.044**	**3.947**	**4.065**	**<.0001**
3	Flycatching	**Horiz. Aspect**	**−2.311**	**0.862**	**−2.683**	**.0073**
	(4 species)	**Tarsus:Wing**	**−29.417**	**5.782**	**−5.088**	**<.0001**
4	Frugivory	**Bill Slenderness**	**−13.874**	**5.342**	**−2.597**	**.0094**
	(4 species)	Mass	−24.994	34.152	−0.7320	.4643

Note

The response variable for each model was the probability of observing the behavior. The prediction from Table 1 that each model addresses is noted in the first column. See text for how the morphological characteristics were calculated. Also shown are the number of species used in each model. Bolded values are significant at the *α* = 0.05 level.

**FIGURE 2:**
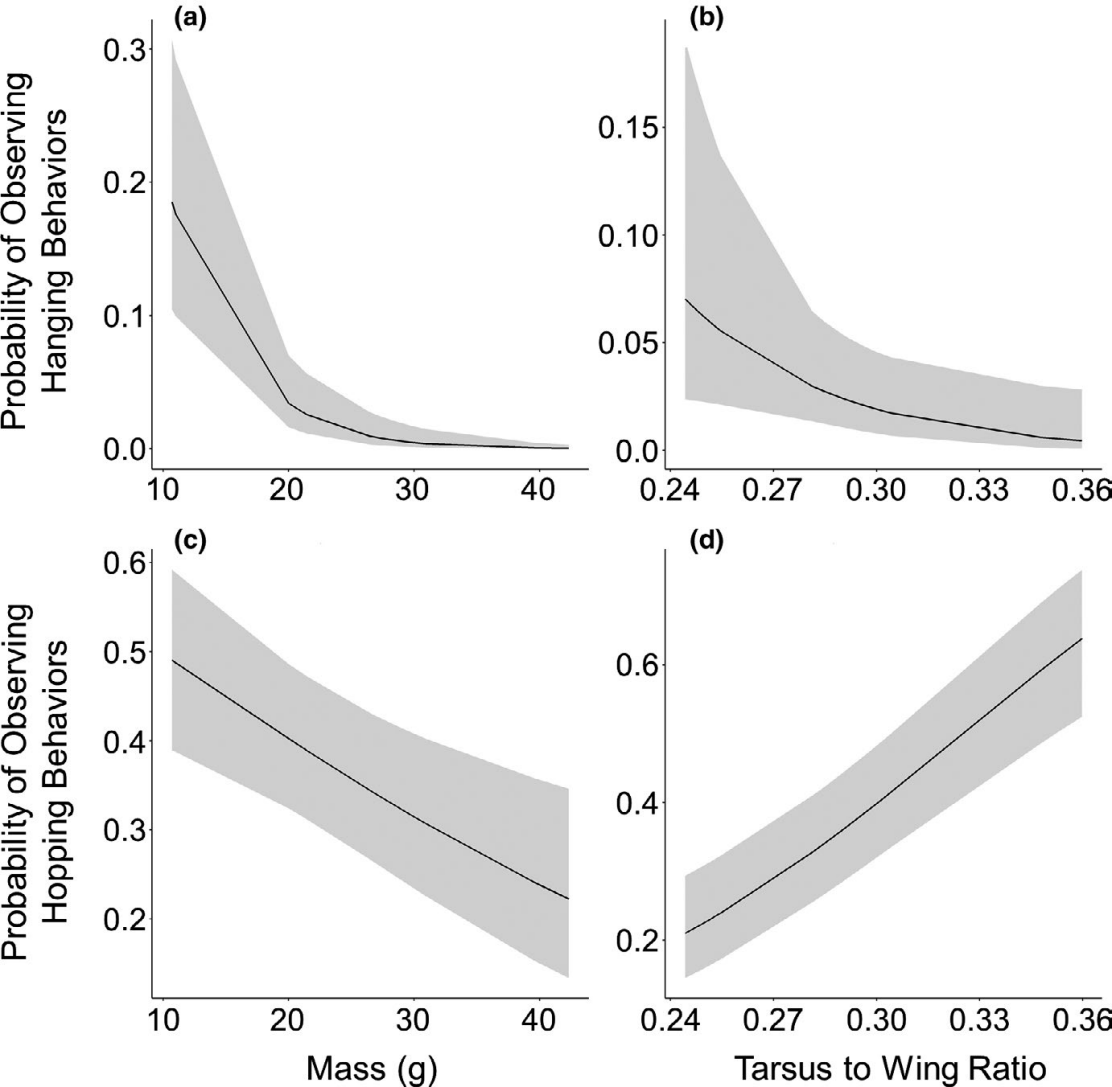
Smaller species with longer tarsi were more likely to be observed hopping while smaller birds with shorter tarsi were more likely to be observed hanging. Shown are the predicted relationships from generalized linear mixed models between the probability of observing hanging behaviors and (a) the average mass of each species at each site and (b) the average tarsus‐to‐wing ratio of each species at each site. Also shown are the predicted relationships between the probability of observing hopping behaviors and (a) the average mass of each species at each site and (b) the average tarsus‐to‐wing ratio of each species at each site. The gray ribbons represent 95% confidence intervals

**FIGURE 3:**
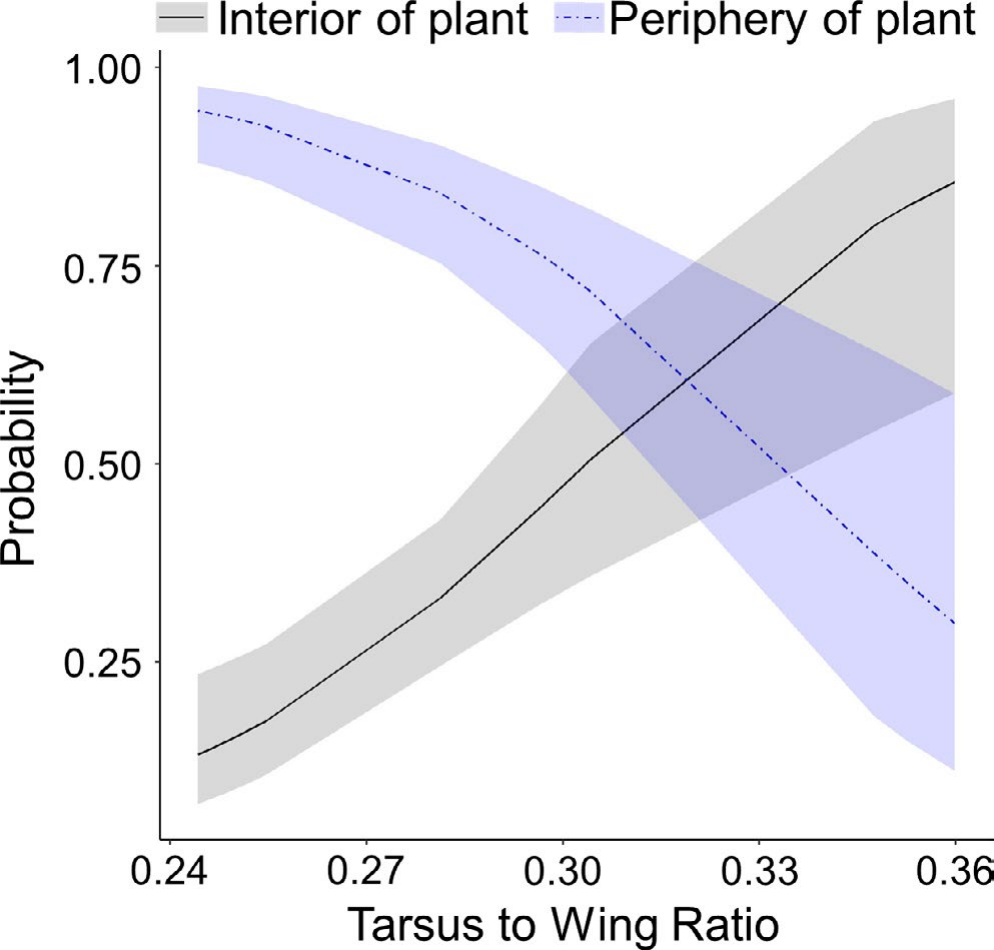
Species with longer tarsi were more likely to be observed in the interior of the plant while species with longer wings were more likely to be observed in the periphery of the plant. Shown are the predicted relationships from generalized linear mixed models between the average tarsus‐to‐wing ratio of each species at each site and the probability of observing the species in the interior of a plant (black line with gray 95% confidence ribbon) and the probability observing the species in the periphery of a plant (blue line with light blue 95% confidence ribbon)

The larger species, in terms of mass, were observed higher in the forest (Table 3). However, the height a species was observed in the forest was not significantly related to the tarsus‐to‐wing ratio even though there was a trend toward species with larger wings in relation to their tarsi being higher in the forest and species with longer tarsi in relation to their wings being lower in the forest (Table 3). The probability of observing hanging behaviors was significantly related to mass, with smaller species, in terms of mass, more likely to hang from branches (Table 4; Figure 2a). Likewise, smaller species were more likely to be observed hopping than larger species (Table 4; Figure 2c). Additionally, species with longer tarsi in relation to their wings were more likely to hop and less likely to hang (Table 4; Figure 2b,d). The species with longer wings in relation to their tarsi were more likely to be observed foraging in the periphery of plants while species with longer tarsi in relation to their wings were more likely to be observed in the interior of plants (Table 4; Figure 3). We did not find a relationship between the species' mass and being observed in the periphery of the tree (Table 4).

**TABLE 3:** Results from the two linear mixed models of the height the birds were observed and their morphology

Prediction	Response variable	Morphology	Estimate	*SE*	*df*	*t*‐Value	*p*‐Value
1	Maximum Height	Tarsus:Wing	−1.956	2.249	9.96	−0.870	.4048
	(5 species)	**Mass**	**12.444**	**5.573**	**129.14**	**2.233**	**.0273**
1	Minimum Height	Tarsus:Wing	−1.685	2.334	11.25	−0.722	.4851
	(5 species)	**Mass**	**17.174**	**5.623**	**158.34**	**3.054**	**.0027**

Note

The response variable was the relative maximum and minimum heights each bird was observed (observed height divided by average canopy height). The prediction from Table 1 that each model addresses is noted in the first column. Also shown are the number of species used in each model. Bolded values are significant at the *α* = 0.05 level.

**TABLE 4:** Results from the generalized linear mixed models of four observed movement behaviors and foraging locations with morphology as the independent variable

Prediction	Response variable	Morphology	Estimate	*SE*	*z*‐Value	*p*‐Value
5	Hanging	**Tarsus:Wing**	**−24.326**	**10.867**	**−2.239**	**.0252**
	(5 species)	**Mass**	**−200.017**	**35.167**	**−5.688**	**<.0001**
6	Hopping	**Tarsus:Wing**	**16.385**	**2.690**	**6.092**	**<.0001**
	(5 species)	**Mass**	**−38.359**	**12.031**	**−3.188**	**.0014**
7	In Periphery	**Tarsus:Wing**	**−32.054**	**7.737**	**−4.143**	**<.0001**
	(5 species)	Mass	−25.943	28.938	−0.896	.3700
7	In Interior	**Tarsus:Wing**	**31.563**	**8.425**	**3.747**	**.0002**
	(5 species)	Mass	41.853	25.071	1.669	.0950
8	On Ground	Tarsus:Wing	29.635	15.615	1.898	.0577
	(5 species)	Mass	74.694	51.657	1.446	.1482

Note

The response variable for each model was the probability of observing the behavior. The prediction from Table 1 that each model addresses is noted in the first column. See text for how the morphological characteristics were calculated. Also shown are the number of species used in each model. Bolded values are significant at the *α* = 0.05 level.

**FIGURE 4:**
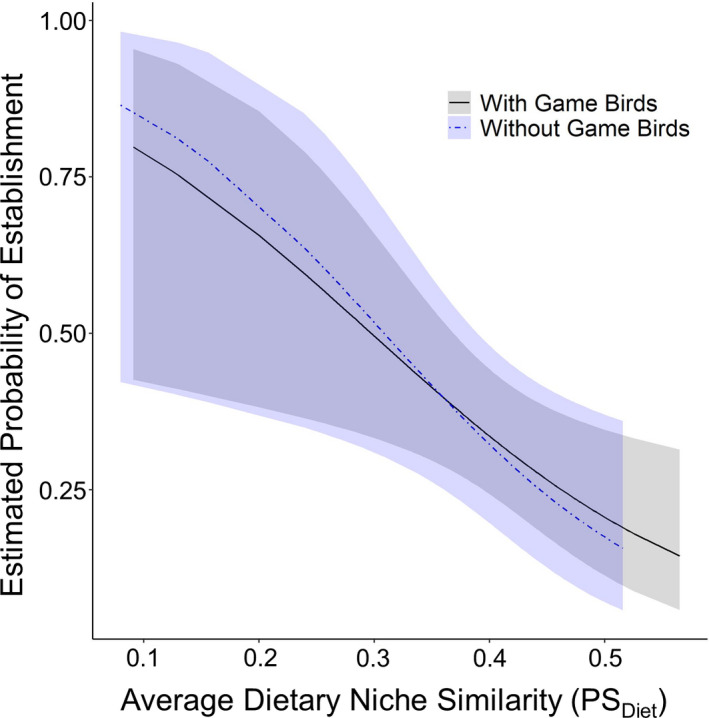
The probability that an introduced bird species established on Oahu was reduced if their dietary niche was more similar on average to the incumbent bird community. Shown are the predicted relationships from generalized linear mixed models between the probability of establishment and the average proportional similarity of diet for an introduced species with (black line with gray 95% confidence ribbon) and without (blue line with light blue 95% confidence ribbon) game birds

An introduced species’ probability of establishing on O‘ahu was negatively related to the average similarity of its diets with the incumbent species (PS_diet_; Table 5; Figure 4) so that species with more similar diets to the incumbent community had a reduced chance of establishing. This result was also found when excluding gamebirds that were introduced for hunting purposes (Table 5; Figure 4). The species richness of the incumbent community was only significantly related to an introduced species probability of establishing when game birds were excluded (Table 5) with species having a smaller chance of establishing if more species were already present in the community. Neither average similarity in foraging strata use distributions (PS_for_) between an introduced species and the incumbent community nor the number of years the species was introduced was significantly related to the probability of establishment (Table 5).

**TABLE 5:** The probability of an introduced species establishing was negatively related to the average similarity of the introduced species' diet resource distribution with the incumbent community

Community	Variable	Estimate	*SE*	*z*	*p*‐Value
All Terrestrial Bird Species	Species Richness	−0.028	0.015	−1.821	.069
	Number of Introduction Years	0.013	0.017	0.730	.465
	**Proportional Similarity of Diet**	**−6.649**	**2.692**	**−2.470**	**.014**
	Proportional Similarity of Foraging Strata Use	1.131	1.341	0.843	.399
Without Game Bird Species	**Species Richness**	**−0.062**	**0.023**	**−2.677**	**.007**
	Number of Introduction Years	0.002	0.023	0.067	.947
	**Proportional Similarity of Diet**	**−8.109**	**3.462**	**−2.342**	**.019**
	Proportional Similarity of Foraging Strata Use	3.891	2.090	1.861	.063

Note

Shown are the result from two binomial generalized linear models with establishment as the response and species' taxonomic family as a random effect with and without game birds. Species richness was the number of species in the incumbent community, and the number of introduction years was the number of years the species was reported to be introduced or escaped. The proportional similarity of diet and forest strata use measures the average similarity between the introduced species' and incumbent community's diet and foraging strata distributions. Bolded values are significant at the *α* = 0.05 level.

## 4 DISCUSSION

We found significant relationships between morphology and ecology within a novel community of bird species. All of the foraging behaviors we observed (frugivory, flycatching, and gleaning) were significantly related to at least one morphological trait. In addition, the movement behaviors, foraging location (i.e., interior vs. exterior of plant), and minimum height in the forest showed significant relationships with morphology. The morphological dissimilarity was also a significant predictor of niche similarity further supporting the conclusion that there is a strong relationship between form and function within the bird community on O‘ahu. Since ecomorphological relationships are thought to be the product of evolutionary adaptation to a species environment (Ricklefs & Miles, 1994), the lack of evolutionary history between these species and the environment on O‘ahu, and the behavioral flexibility of introduced species (Wright et al., 2010), may lead to the expectation that ecomorphological relationships may be lacking or modified. Since we found the majority of ecological characteristics we investigated were related to at least one morphological trait in the same direction as reported relationships in the literature, we hypothesize that ecological fitting during the invasion process gave rise to these relationships and to the structure of the novel bird community on O‘ahu. This hypothesis is further supported by our finding that the probability of establishment increased the more dissimilar a species’ dietary niche is from the incumbent community. Similarly, Vizentin‐Bugoni et al. (2019) hypothesized that ecological fitting may have led to the complex and stable structure of the seed dispersal networks on the island. However, because rapid morphological change has also been described in our focal species (except WRSH; Gleditsch & Sperry, 2019), the influence of evolution after their introduction on these relationships cannot be ignored.

The relationships we found in this novel system largely corroborate what has been previously described in the literature for native‐dominated systems. Species that depend more on wing‐aided movements for their foraging (e.g., flycatching) tend to have shorter legs due to the beneficial reduction in drag and weight during flight (Leisler & Winkler, 1985). Our results show similar trends with species with shorter tarsi relative to their wings more likely to be observed flycatching and/or foraging in the periphery of plants, both behaviors that rely more on flight than other behaviors. Conversely, we found species with longer tarsi relative to their wings were more likely to engage in hopping movements and forage in the interior of the plants. Indeed, Zeffer et al. (2003) suggested that species that rely more on hopping for movement should have longer tarsi. The significant positive relationship between the tarsus‐to‐wing ratio and gleaning also supports this as longer tarsi would aid in hopping from branch to branch during foraging—a behavior often observed with gleaning birds (Holmes & Recher, 1986; Robinson & Holmes, 1982). Also, in agreement with previous studies (see Zeffer et al., 2003), we found that smaller species with larger tarsi in relation to wing length were more likely to be observed hanging during foraging. On O‘ahu, this relationship is likely driven by JAWE, which are the smallest of the focal species (Appendix S4) and were 4.5 times more likely to be observed hanging than the next most likely species, RWBU (Appendix S5).

Much of the previous research on bill ecomorphology has been focused on granivores (e.g., Herrel et al., 2005; Price, 1987) or species with specialized feeding modes (e.g., Maglianesi et al., 2014). Relationships among species that eat invertebrates and fruit may have weaker relationships than those that eat seeds due to the increased mechanical stress on granivorous species’ bills (Lederer, 1975; Leisler & Winkler, 1985). Still, we found significant relationships between bill morphology and the probability of observing three common foraging behaviors. It has been suggested that more robust bills (wider and deeper) are characteristic of a more generalized diet and increased frugivory (Moermond & Denslow, 1985; Snow & Snow, 1988). We found a similar result with the probability of observing frugivory increasing with bill robustness (i.e., negative relationship with bill slenderness; Figure 1b). Wider bills are often considered to be characteristic of birds that flycatch during foraging while slender bills are often considered characteristic of birds that glean (Leisler & Winkler, 1985). The relationships that we found support this conclusion. We observed species with wider bills were more likely to be observed flycatching (i.e., negative relationship with the horizontal bill aspect ratio) while birds with more slender bills were more likely to be observed gleaning (i.e., positive relationship with bill slenderness; Figure 1a). However, within the focal community of this study JAWE are an outlier in their bill morphology having the slenderest bills of our focal species (Appendix S4), potentially unduly influencing the models.

Although most of our results matched those predicted by the literature, several relationships we analyzed were nonsignificant or in contrast to the literature. This is likely due to the relatively small number of species included in our analyses, all belonging to similar foraging guilds. For instance, we did not find a relationship between the tarsus‐to‐wing ratio and the probability of observing a bird on the ground which contrasts with the expectation that species with larger tarsi tend to forage on the ground (Zeffer et al., 2003). The theorized relationship between foraging on the ground and tarsus length stems from the increased step length produced by larger tarsi making movement along the ground more efficient (Zeffer et al., 2003). The species in our study are mainly arboreal and belong to the same foraging guild while the studies that show a relationship between foraging on the ground and tarsus length typically include multiple guilds (Leisler & Winkler, 1985; Zeffer et al., 2003). Additionally, our results suggest larger birds are more likely to forage higher in the forest. This is counter to previous studies documenting that smaller birds (based on body size) are more likely to forage higher in the canopy (Cody & Mooney, 1978; Forstmeier & Keßler, 2001). Because we used mist‐nets, the observed relationship between foraging height and morphology may have been biased due to only catching individuals lower in the forest, and therefore with morphologies associated with foraging lower. However, the largest species in our study is the RVBU was almost exclusively observed in or on top of the forest canopy and is likely driving this result. It is possible that the inclusion of more species (e.g., game birds) across foraging guilds (e.g., granivores) may further elucidate ecomorphological relationships that better match those proposed by the literature. Our results showing that species pairs with more different morphologies have more different foraging niches suggest that there is a strong relationship between ecological niche and morphology, even when looking across guilds.

It is common in species introduced to islands to expand their niche by incorporating more resource types into their diet or occupying a wider range of environments due to ecological release (MacArthur et al., 1972). Four of these species exhibited morphological shifts that may support the notion that they now occupy a more generalized niche (i.e., more robust bills; Gleditsch & Sperry, 2019), and, interestingly, the morphological changes observed in Gleditsch and Sperry (2019) align well with the ecomorphological relationships we observed here. In that study, the bird species that exhibited the largest shifts toward more robust bills were the species in this study that exhibited the most frugivory (RBLE, RVBU, and RWBU; Appendix S5 and see Vizentin‐Bugoni et al., 2019). Additionally, the species that had shorter wings on O‘ahu included the species that were the least likely to be observed flycatching and were the species observed lower in the forest (JAWE, RBLE, and RWBU; Appendix S5). RVBU and RWBU had similar morphologies in this study (except RVBU are larger) and are found in the same types of habitat, but they occupied different forest strata with RWBU being observed lower in the forest more often than RVBU (Appendix S5). Gleditsch and Sperry (2019) found that RWBU had shorter wings on O‘ahu, and thus, they may have shifted their behaviors to limit the ecological overlap with RVBU creating evolutionary pressures selecting for shorter wings. However, the detailed ecological data for these species in their native ranges are lacking, and therefore, we cannot determine the causal relationships between evolutionary and ecological processes. From other accounts, the two species seem to have very similar generalized foraging ecologies in the native range but occupy different habitats with RWBU confined to wetter, more evergreen forests than RVBU (Fishpool and Tobias, 2005).

We found that the probability of establishment was negatively related to the average similarity of dietary niche between the introduced species and the incumbent community, suggesting that the only species that were able to establish on O‘ahu were those that had ecologies different enough from the incumbent community that they were able to fill the available niche space. This results the fact that the majority of ecomorphological relationships we found match those proposed by the literature, and the negative trend between morphological and foraging niche similarity suggests that ecological fitting was important in the bird community assembly of this novel system. Additionally, supporting this conclusion, of the 47 extant terrestrial bird species (excluding rails, herons, and sandpipers) on O‘ahu, only four genera have more than one species (Pyle & Pyle, 2017), suggesting phylogenic overdispersion. Congeneric species may be more likely to have similar morphology and overlapping niches increasing their competition and leading to only one of those species successfully establishing. However, if the species are morphologically distinct, they may have ecologies that differ enough to coexist. Indeed, when examining the finch species that have been introduced onto O‘ahu, Moulton and Lockwood (1992) found that the fifteen species that have successfully established were morphologically overdispersed when compared to pools of other species. This result was upheld when they then examined finch species found in a single type of habitat.

The commonness of significant relationships between ecology and morphology in this novel community and the fact that species with more dissimilar morphologies have more different ecology suggests a strong relationship between form and function. Evolutionary processes, while still likely acting on these species, appear to be secondary to ecological fitting in structuring the novel bird community, fine‐tuning the form‐function relationships. Still, since the relationships between form and function were conserved in the novel bird community on O‘ahu, evolutionary process, such as those previously found in Hawai‘i, may influence the ecological function of birds species on the island. Changes in the ability of the introduced species to fulfill their ecological function could mitigate or exasperate the effects of the introduction and alter the functioning and stability of the novel systems now present in many regions of the world. Although we see these relationships in O‘ahu, novel systems, almost by their very nature, are unpredictable. O‘ahu presents an extreme case of novel systems, and a more global look at ecomorphological relationships, across a gradient of native to non‐native communities, would be required to understand the potential of rapid evolutionary changes to influence species’ ecological functioning.

## CONFLICT OF INTERESTS

The authors have no competing interests regarding this research.

## AUTHOR CONTRIBUTION

**Jason M. Gleditsch:** Conceptualization (equal); Data curation (lead); Formal analysis (lead); Investigation (lead); Methodology (lead); Visualization (lead); Writing‐original draft (lead); Writing‐review & editing (equal). **Jinelle H. Sperry:** Conceptualization (equal); Formal analysis (supporting); Funding acquisition (lead); Investigation (supporting); Methodology (supporting); Project administration (equal); Resources (lead); Supervision (lead); Writing‐original draft (supporting); Writing‐review & editing (equal).

## Supporting information

Supplementary Material

## Data Availability

The data used for the research presented in this manuscript are available on Dryad https://doi.org/10.5061/dryad.4b8gtht9z.
