# Supplementary material for: Ecomorphological relationships and invasion history of non‐native terrestrial bird species on O‘ahu, Hawai‘i, suggest ecological fitting during novel community assembly
Source: Ecol Evol. 2020 Oct 3;10(21):12157–69. doi: 10.1002/ece3.6843 (PMC7663968; doi:10.1002/ece3.6843)
Supplement: Supplementary file 1 — Supplementary Material [file ECE3-10-12157-s001.docx]

**Appendix s1**: Diet resource distribution data obtained from EltonTraits 1.0

The diet resource distribution for each species listed in Pyle and Pyle (2017) as resident, extinct, or introduced but failed to establish. Shown are the estimated proportion of diet that each resource type comprises (in percent). Invertebrate and vertebrate resource types are abbreviated Invert. and Vert., respectively. Estimates taken from Wilman et al. (2014).

| Species | Invert. | Vert. | Scavenge | Fruit | Nectar | Seed | Plant |
| --- | --- | --- | --- | --- | --- | --- | --- |
| *Acridotheres tristis* | 40 | 30 | 0 | 10 | 10 | 10 | 0 |
| *Aerodramus bartschi* | 100 | 0 | 0 | 0 | 0 | 0 | 0 |
| *Agapornis personatus* | 0 | 0 | 0 | 0 | 0 | 100 | 0 |
| *Agapornis roseicollis* | 0 | 0 | 0 | 0 | 0 | 70 | 30 |
| *Akialoa ellisiana* | NA | 0 | NA | NA | NA | NA | NA |
| *Alauda arvensis* | 40 | 0 | 0 | 0 | 0 | 40 | 20 |
| *Amandava amandava* | 20 | 0 | 0 | 0 | 0 | 80 | 0 |
| *Amazona aestiva* | 0 | 0 | 0 | 50 | 0 | 50 | 0 |
| *Amazona albifrons* | 0 | 0 | 0 | 20 | 0 | 50 | 30 |
| *Amazona autumnalis* | 0 | 0 | 0 | 100 | 0 | 0 | 0 |
| *Amazona oratrix* | 0 | 0 | 0 | 40 | 0 | 30 | 30 |
| *Amazona viridigenalis* | 0 | 0 | 0 | 30 | 0 | 30 | 40 |
| *Ara ararauna* | 0 | 0 | 0 | 30 | 10 | 30 | 30 |
| *Ara macao* | 0 | 0 | 0 | 20 | 20 | 30 | 30 |
| *Aratinga jandaya* | 0 | 0 | 0 | 80 | 0 | 20 | 0 |
| *Aratinga nenday* | 0 | 0 | 0 | 30 | 0 | 30 | 40 |
| *Asio flammeus* | 0 | 100 | 0 | 0 | 0 | 0 | 0 |
| *Brotogeris jugularis* | 0 | 0 | 0 | 30 | 20 | 30 | 20 |
| *Buteogallus meridionalis* | 20 | 80 | 0 | 0 | 0 | 0 | 0 |
| *Cacatua alba* | 10 | 0 | 0 | 0 | 0 | 90 | 0 |
| *Cacatua galerita* | 0 | 0 | 0 | 40 | 0 | 40 | 20 |
| *Cacatua goffiniana* | 10 | 0 | 0 | 20 | 0 | 60 | 10 |
| *Cacatua moluccensis* | 0 | 0 | 0 | 50 | 0 | 50 | 0 |
| *Cacatua sulphurea* | 0 | 0 | 0 | 50 | 0 | 50 | 0 |
| *Cardinalis cardinalis* | 20 | 0 | 0 | 10 | 0 | 0 | 70 |
| *Cettia diphone* | 100 | 0 | 0 | 0 | 0 | 0 | 0 |
| *Chalcophaps indica* | 20 | 0 | 0 | 40 | 0 | 40 | 0 |
| *Chasiempis ibidis* | 100 | 0 | 0 | 0 | 0 | 0 | 0 |
| *Chlorodrepanis flavus* | 30 | 0 | 0 | 30 | 40 | 0 | 0 |
| *Chrysolophus amherstiae* | 40 | 0 | 0 | 10 | 0 | 0 | 50 |
| *Chrysolophus pictus* | 10 | 0 | 0 | 0 | 0 | 0 | 90 |
| *Colinus virginianus* | 0 | 0 | 0 | 0 | 0 | 70 | 30 |
| *Columba livia* | 10 | 0 | 0 | 0 | 0 | 60 | 30 |

**Appendix s1 (continued)**:

| Species | | Invert. | | Vert. | | Scavenge | | Fruit | | Nectar | | Seed | | Plant | |
| --- | --- | --- | --- | --- | --- | --- | --- | --- | --- | --- | --- | --- | --- | --- | --- |
| *Copsychus malabaricus* | | 70 | | 0 | | 0 | | 30 | | 0 | | 0 | | 0 | |
| *Copsychus saularis* | | 70 | | 10 | | 0 | | 10 | | 10 | | 0 | | 0 | |
| *Coturnix chinensis* | | 30 | | 0 | | 0 | | 0 | | 0 | | 40 | | 30 | |
| *Coturnix japonica* | | 30 | | 0 | | 0 | | 0 | | 0 | | 0 | | 70 | |
| *Cyanoptila cyanomelana* | | 90 | | 0 | | 0 | | 10 | | 0 | | 0 | | 0 | |
| *Cyanoramphus novaezelandiae* | | 10 | | 0 | | 0 | | 30 | | 0 | | 30 | | 30 | |
| ***Drepanis coccinea*** | | **20** | | **0** | | **0** | | **0** | | **80** | | **0** | | **0** | |
| *Eclectus roratus* | | 0 | | 0 | | 0 | | 40 | | 0 | | 30 | | 30 | |
| *Erithacus akahige* | | 40 | | 20 | | 10 | | 20 | | 0 | | 10 | | 0 | |
| *Erithacus komadori* | | 40 | | 20 | | 10 | | 20 | | 0 | | 10 | | 0 | |
| *Estrilda astrild* | | 0 | | 0 | | 0 | | 0 | | 0 | | 100 | | 0 | |
| *Estrilda caerulescens* | | 0 | | 0 | | 0 | | 0 | | 0 | | 100 | | 0 | |
| *Estrilda melpoda* | | 40 | | 0 | | 0 | | 0 | | 0 | | 60 | | 0 | |
| *Euodice cantans* | | 10 | | 0 | | 0 | | 0 | | 0 | | 90 | | 0 | |
| *Euplectis afer* | | 20 | | 0 | | 0 | | 0 | | 0 | | 80 | | 0 | |
| *Euplectis franciscanus* | | 20 | | 0 | | 0 | | 0 | | 0 | | 80 | | 0 | |
| *Euplectis orix* | | 50 | | 0 | | 0 | | 0 | | 0 | | 50 | | 0 | |
| *Eupsittula canicularis* | | 0 | | 0 | | 0 | | 50 | | 0 | | 50 | | 0 | |
| *Ficedula narcissina* | | 80 | | 0 | | 0 | | 20 | | 0 | | 0 | | 0 | |
| *Francolinus erckelii* | | 30 | | 0 | | 0 | | 0 | | 0 | | 0 | | 70 | |
| *Francolinus francolinus* | | 10 | | 0 | | 0 | | 0 | | 20 | | 20 | | 50 | |
| *Francolinus pondicerianus* | | 10 | | 0 | | 0 | | 20 | | 0 | | 30 | | 40 | |
| *Gallus gallus* | | 30 | | 0 | | 0 | | 20 | | 0 | | 20 | | 30 | |
| *Garrulax canorus* | | 70 | | 0 | | 0 | | 10 | | 0 | | 10 | | 10 | |
| *Garrulax chinensis* | | 70 | | 0 | | 0 | | 0 | | 0 | | 10 | | 20 | |
| *Garrulax leucolophus* | | 60 | | 10 | | 0 | | 10 | | 10 | | 10 | | 0 | |
| *Geopelia cuneata* | | 10 | | 0 | | 0 | | 0 | | 0 | | 70 | | 20 | |
| *Geopelia humeralis* | | 0 | | 0 | | 0 | | 0 | | 0 | | 40 | | 60 | |
| *Geopelia placida* | | 20 | | 0 | | 0 | | 0 | | 0 | | 80 | | 0 | |
| *Geopelia striata* | | 20 | | 0 | | 0 | | 0 | | 0 | | 80 | | 0 | |
| *Gracula religiosa* | | 20 | | 10 | | 0 | | 60 | | 10 | | 0 | | 0 | |
| *Gracupica nigricollis* | | 70 | | 0 | | 0 | | 0 | | 0 | | 30 | | 0 | |
| *Grallina cyanoleuca* | | 70 | | 20 | | 0 | | 0 | | 10 | | 0 | | 0 | |
| *Gubernatrix cristata* | | 0 | | 0 | | 0 | | 0 | | 0 | | 100 | | 0 | |
| *Haemorhous mexicanus* | 10 | | 0 | | 0 | | 30 | | 0 | | 30 | | 30 | |  |
| ***Hemignathus lucidus*** | | **100** | | **0** | | **0** | | **0** | | **0** | | **0** | | **0** | |
| ***Himatione sanguinea*** | | **20** | | **0** | | **0** | | **0** | | **80** | | **0** | | **0** | |
| *Lagonosticta rubricata* | | 20 | | 0 | | 0 | | 0 | | 0 | | 80 | | 0 | |

**Appendix s1 (continued)**:

| Species | Invert. | Vert. | Scavenge | Fruit | Nectar | Seed | Plant |
| --- | --- | --- | --- | --- | --- | --- | --- |
| *Leiothrix lutea* | 80 | 0 | 0 | 10 | 0 | 10 | 0 |
| *Leucopsar rothschildi* | 30 | 0 | 0 | 70 | 0 | 0 | 0 |
| *Lonchura atricapilla* | 0 | 0 | 0 | 0 | 0 | 100 | 0 |
| *Lonchura malacca* | 0 | 0 | 0 | 0 | 0 | 100 | 0 |
| *Lonchura oryzivora* | 10 | 0 | 0 | 0 | 0 | 90 | 0 |
| *Lonchura punctulata* | 10 | 0 | 0 | 20 | 0 | 50 | 20 |
| *Lophura leucomelanos* | 30 | 10 | 0 | 30 | 0 | 30 | 0 |
| *Lophura nycthemera* | 30 | 0 | 0 | 30 | 0 | 40 | 0 |
| *Loxops wolstenholmei* | NA | 0 | NA | NA | NA | NA | NA |
| *Meleagris gallopavo* | 20 | 0 | 0 | 20 | 0 | 20 | 40 |
| *Melopsittacus undulatus* | 0 | 0 | 0 | 0 | 0 | 100 | 0 |
| *Mimus polyglottos* | 50 | 0 | 0 | 50 | 0 | 0 | 0 |
| *Moho apicalis* | NA | 0 | NA | NA | NA | NA | NA |
| *Myadestes woahensis* | NA | 0 | NA | NA | NA | NA | NA |
| *Myiopsitta monachus* | 10 | 0 | 0 | 30 | 0 | 30 | 30 |
| *Nectariniidae sp.* | 65 | 0 | 0 | 20 | 10 | 5 | 0 |
| *Numida meleagris* | 30 | 0 | 0 | 10 | 0 | 20 | 40 |
| *Nymphicus hollandicus* | 0 | 0 | 0 | 0 | 0 | 100 | 0 |
| *Ocyphaps lophotes* | 10 | 0 | 0 | 0 | 0 | 50 | 40 |
| *Oreortyx pictus* | 0 | 0 | 0 | 20 | 0 | 40 | 40 |
| *Oriolus chinensis* | 20 | 20 | 0 | 60 | 0 | 0 | 0 |
| *Oriolus xanthornus* | 20 | 0 | 0 | 70 | 10 | 0 | 0 |
| *Paroaria coronata* | 60 | 0 | 0 | 20 | 0 | 0 | 20 |
| *Paroaria dominicana* | 60 | 0 | 0 | 20 | 0 | 0 | 20 |
| *Paroaria gularis* | 60 | 0 | 0 | 20 | 0 | 0 | 20 |
| *Paroreomyza maculata* | 80 | 0 | 0 | 0 | 20 | 0 | 0 |
| *Parus varius* | 70 | 0 | 0 | 10 | 0 | 20 | 0 |
| *Passer domesticus* | 10 | 0 | 0 | 0 | 0 | 60 | 30 |
| *Passerina ciris* | 0 | 0 | 0 | 0 | 0 | 100 | 0 |
| *Passerina cyanea* | 0 | 0 | 0 | 0 | 0 | 100 | 0 |
| *Passerina leclancherii* | 30 | 0 | 0 | 0 | 0 | 70 | 0 |
| *Pavo cristatus* | 30 | 10 | 0 | 0 | 0 | 40 | 20 |
| *Perdix perdix* | 30 | 0 | 0 | 0 | 0 | 30 | 40 |
| *Phaps chalcoptera* | 10 | 0 | 0 | 0 | 0 | 70 | 20 |
| *Phasianus colchicus* | 10 | 0 | 0 | 30 | 0 | 30 | 30 |
| *Ploceus philippinus* | 20 | 10 | 0 | 0 | 0 | 70 | 0 |
| *Poicephalus senegalus* | 0 | 0 | 0 | 40 | 0 | 30 | 30 |

**Appendix s1 (continued)**:

| Species | Invert. | Vert. | Scavenge | Fruit | Nectar | Seed | Plant |
| --- | --- | --- | --- | --- | --- | --- | --- |
| *Probosciger aterrimus* | 0 | 0 | 0 | 40 | 0 | 30 | 30 |
| *Psittacula cyanocephala* | 0 | 0 | 0 | 80 | 0 | 20 | 0 |
| *Psittacula krameri* | 0 | 0 | 0 | 50 | 10 | 20 | 20 |
| *Psitticara acuticaudata* | 0 | 0 | 0 | 50 | 0 | 50 | 0 |
| *Psitticara erythrogenys* | 0 | 0 | 0 | 60 | 0 | 0 | 40 |
| *Psittirostra psittacea* | 20 | 0 | 0 | 70 | 0 | 0 | 10 |
| *Pycnonotus cafer* | 20 | 10 | 0 | 20 | 20 | 10 | 20 |
| *Pycnonotus jocosus* | 20 | 0 | 0 | 20 | 20 | 20 | 20 |
| *Rhipidura leucophrys* | 70 | 20 | 0 | 0 | 0 | 10 | 0 |
| *Rollulus rouloul* | 40 | 0 | 0 | 30 | 0 | 30 | 0 |
| *Serinus leucopygius* | 0 | 0 | 0 | 0 | 0 | 80 | 20 |
| *Serinus mozambicus* | 10 | 0 | 0 | 0 | 0 | 50 | 40 |
| *Sicalis flaveola* | 0 | 0 | 0 | 0 | 0 | 100 | 0 |
| *Spilopelia chinensis* | 0 | 0 | 0 | 0 | 0 | 100 | 0 |
| *Stagonopleura guttata* | 20 | 0 | 0 | 0 | 0 | 80 | 0 |
| *Streptopelia decaocto* | 10 | 0 | 0 | 30 | 0 | 40 | 20 |
| *Streptopelia roseogrisea* | 10 | 0 | 0 | 0 | 0 | 70 | 20 |
| *Syrmaticus reevesii* | 10 | 0 | 0 | 0 | 0 | 40 | 50 |
| *Syrmaticus soemmerringii* | 50 | 0 | 0 | 0 | 0 | 50 | 0 |
| *Taeniopygia guttata* | 0 | 0 | 0 | 0 | 0 | 100 | 0 |
| *Tiaris olivaceus* | 0 | 0 | 0 | 0 | 0 | 90 | 10 |
| *Tympanuchus cupido* | 20 | 0 | 0 | 10 | 0 | 50 | 20 |
| *Tyto alba* | 10 | 90 | 0 | 0 | 0 | 0 | 0 |
| *Uraeginthus angolensis* | 30 | 0 | 0 | 0 | 0 | 70 | 0 |
| *Uraeginthus cyanocephalus* | 20 | 0 | 0 | 0 | 0 | 80 | 0 |
| *Urocissa erythroryncha* | 40 | 40 | 10 | 10 | 0 | 0 | 0 |
| *Vidua chalybeata* | 20 | 10 | 0 | 0 | 0 | 70 | 0 |
| *Vidua macroura* | 20 | 0 | 0 | 0 | 0 | 80 | 0 |
| *Zenaida macroura* | 0 | 0 | 0 | 0 | 0 | 90 | 10 |
| *Zosterops japonicus* | 40 | 0 | 0 | 30 | 30 | 0 | 0 |

**Appendix s2**: Foraging strata use distribution data obtained from EltonTraits 1.0

The foraging strata use distribution for each species listed in Pyle and Pyle (2017) as resident, extinct, or introduced but failed to establish. Shown are the estimated proportion of foraging time that four forest strata are used (in percent). Estimates taken from Wilman et al. (2014).

| Species | Ground | Understory | Midstory | Canopy | Aerial |
| --- | --- | --- | --- | --- | --- |
| *Acridotheres tristis* | 70 | 20 | 10 | 0 | 0 |
| *Aerodramus bartschi* | 0 | 0 | 0 | 20 | 80 |
| *Agapornis personatus* | 60 | 40 | 0 | 0 | 0 |
| *Agapornis roseicollis* | 50 | 50 | 0 | 0 | 0 |
| *Akialoa ellisiana* | NA | NA | NA | NA | NA |
| *Alauda arvensis* | 100 | 0 | 0 | 0 | 0 |
| *Amandava amandava* | 50 | 50 | 0 | 0 | 0 |
| *Amazona aestiva* | 0 | 30 | 40 | 30 | 0 |
| *Amazona albifrons* | 0 | 50 | 50 | 0 | 0 |
| *Amazona autumnalis* | 0 | 20 | 40 | 40 | 0 |
| *Amazona oratrix* | 33 | 33 | 33 | 0 | 0 |
| *Amazona viridigenalis* | 20 | 20 | 40 | 20 | 0 |
| *Ara ararauna* | 0 | 0 | 60 | 40 | 0 |
| *Ara macao* | 0 | 0 | 60 | 40 | 0 |
| *Aratinga jandaya* | 0 | 33 | 33 | 33 | 0 |
| *Aratinga nenday* | 100 | 0 | 0 | 0 | 0 |
| *Asio flammeus* | 90 | 0 | 10 | 0 | 0 |
| *Brotogeris jugularis* | 0 | 0 | 60 | 40 | 0 |
| *Buteogallus meridionalis* | 100 | 0 | 0 | 0 | 0 |
| *Cacatua alba* | 10 | 10 | 40 | 40 | 0 |
| *Cacatua galerita* | 40 | 10 | 30 | 20 | 0 |
| *Cacatua goffiniana* | 50 | 50 | 0 | 0 | 0 |
| *Cacatua moluccensis* | 20 | 30 | 30 | 20 | 0 |
| *Cacatua sulphurea* | 0 | 0 | 60 | 40 | 0 |
| *Cardinalis cardinalis* | 40 | 20 | 20 | 20 | 0 |
| *Cettia diphone* | 50 | 50 | 0 | 0 | 0 |
| *Chalcophaps indica* | 70 | 10 | 10 | 10 | 0 |
| *Chasiempis ibidis* | 20 | 20 | 60 | 0 | 0 |
| *Chlorodrepanis flavus* | 10 | 40 | 50 | 0 | 0 |
| *Chrysolophus amherstiae* | 100 | 0 | 0 | 0 | 0 |
| *Chrysolophus pictus* | 100 | 0 | 0 | 0 | 0 |
| *Colinus virginianus* | 100 | 0 | 0 | 0 | 0 |
| *Columba livia* | 80 | 20 | 0 | 0 | 0 |

**Appendix s2 (continued)**:

| Species | Ground | | Understory | | Midstory | | Canopy | | Aerial | |  |
| --- | --- | --- | --- | --- | --- | --- | --- | --- | --- | --- | --- |
| *Copsychus malabaricus* | | 60 | | 40 | | 0 | | 0 | | 0 | |
| *Copsychus saularis* | | 80 | | 20 | | 0 | | 0 | | 0 | |
| *Coturnix chinensis* | | 100 | | 0 | | 0 | | 0 | | 0 | |
| *Coturnix japonica* | | 100 | | 0 | | 0 | | 0 | | 0 | |
| *Cyanoptila cyanomelana* | | 0 | | 20 | | 40 | | 40 | | 0 | |
| *Cyanoramphus novaezelandiae* | | 20 | | 40 | | 40 | | 0 | | 0 | |
| *Drepanis coccinea* | | 100 | | 0 | | 0 | | 0 | | 0 | |
| *Eclectus roratus* | | 0 | | 40 | | 40 | | 20 | | 0 | |
| *Erithacus akahige* | | 50 | | 50 | | 0 | | 0 | | 0 | |
| *Erithacus komadori* | | 100 | | 0 | | 0 | | 0 | | 0 | |
| *Estrilda astrild* | | 50 | | 50 | | 0 | | 0 | | 0 | |
| *Estrilda caerulescens* | | 33 | | 33 | | 33 | | 0 | | 0 | |
| *Estrilda melpoda* | | 50 | | 50 | | 0 | | 0 | | 0 | |
| *Euodice cantans* | | 80 | | 20 | | 0 | | 0 | | 0 | |
| *Euplectis afer* | | 80 | | 20 | | 0 | | 0 | | 0 | |
| *Euplectis franciscanus* | | 0 | | 20 | | 80 | | 0 | | 0 | |
| *Euplectis orix* | | 0 | | 0 | | 20 | | 80 | | 0 | |
| *Eupsittula canicularis* | | 0 | | 50 | | 50 | | 0 | | 0 | |
| *Ficedula narcissina* | | 0 | | 33 | | 33 | | 33 | | 0 | |
| *Francolinus erckelii* | | 60 | | 40 | | 0 | | 0 | | 0 | |
| *Francolinus francolinus* | | 100 | | 0 | | 0 | | 0 | | 0 | |
| *Francolinus pondicerianus* | | 100 | | 0 | | 0 | | 0 | | 0 | |
| *Gallus gallus* | | 100 | | 0 | | 0 | | 0 | | 0 | |
| *Garrulax canorus* | | 100 | | 0 | | 0 | | 0 | | 0 | |
| *Garrulax chinensis* | | 0 | | 50 | | 50 | | 0 | | 0 | |
| *Garrulax leucolophus* | | 100 | | 0 | | 0 | | 0 | | 0 | |
| *Geopelia cuneata* | | 100 | | 0 | | 0 | | 0 | | 0 | |
| *Geopelia humeralis* | | 100 | | 0 | | 0 | | 0 | | 0 | |
| *Geopelia placida* | | 100 | | 0 | | 0 | | 0 | | 0 | |
| *Geopelia striata* | | 100 | | 0 | | 0 | | 0 | | 0 | |
| *Gracula religiosa* | | 0 | | 30 | | 40 | | 30 | | 0 | |
| *Gracupica nigricollis* | | 80 | | 20 | | 0 | | 0 | | 0 | |
| *Grallina cyanoleuca* | | 0 | | 100 | | 0 | | 0 | | 0 | |
| *Gubernatrix cristata* | | 60 | | 30 | | 10 | | 0 | | 0 | |
| *Haemorhous mexicanus* | | 33 | | 33 | | 33 | | 0 | | 0 | |
| *Hemignathus lucidus* | | 0 | | 50 | | 50 | | 0 | | 0 | |
| *Himatione sanguinea* | | 100 | | 0 | | 0 | | 0 | | 0 | |
| *Lagonosticta rubricata* | | 100 | | 0 | | 0 | | 0 | | 0 | |

**Appendix s2 (continued)**:

| Species | Ground | Understory | Midstory | Canopy | Aerial |
| --- | --- | --- | --- | --- | --- |
| *Leiothrix lutea* | 40 | 40 | 20 | 0 | 0 |
| *Leucopsar rothschildi* | 20 | 40 | 40 | 0 | 0 |
| *Lonchura atricapilla* | 60 | 40 | 0 | 0 | 0 |
| *Lonchura malacca* | 100 | 0 | 0 | 0 | 0 |
| *Lonchura oryzivora* | 50 | 50 | 0 | 0 | 0 |
| *Lonchura punctulata* | 50 | 50 | 0 | 0 | 0 |
| *Lophura leucomelanos* | 100 | 0 | 0 | 0 | 0 |
| *Lophura nycthemera* | 100 | 0 | 0 | 0 | 0 |
| *Loxops wolstenholmei* | NA | NA | NA | NA | NA |
| *Meleagris gallopavo* | 90 | 10 | 0 | 0 | 0 |
| *Melopsittacus undulatus* | 60 | 40 | 0 | 0 | 0 |
| *Mimus polyglottos* | 70 | 30 | 0 | 0 | 0 |
| *Moho apicalis* | NA | NA | NA | NA | NA |
| *Myadestes woahensis* | NA | NA | NA | NA | NA |
| *Myiopsitta monachus* | 20 | 20 | 40 | 20 | 0 |
| *Nectariniidae sp.* | 0 | 35 | 40 | 25 | 0 |
| *Numida meleagris* | 100 | 0 | 0 | 0 | 0 |
| *Nymphicus hollandicus* | 60 | 40 | 0 | 0 | 0 |
| *Ocyphaps lophotes* | 100 | 0 | 0 | 0 | 0 |
| *Oreortyx pictus* | 80 | 20 | 0 | 0 | 0 |
| *Oriolus chinensis* | 20 | 10 | 30 | 40 | 0 |
| *Oriolus xanthornus* | 20 | 10 | 30 | 40 | 0 |
| *Paroaria coronata* | 20 | 80 | 0 | 0 | 0 |
| *Paroaria dominicana* | 20 | 80 | 0 | 0 | 0 |
| *Paroaria gularis* | 30 | 60 | 10 | 0 | 0 |
| *Paroreomyza maculata* | 0 | 30 | 60 | 10 | 0 |
| *Parus varius* | 0 | 0 | 40 | 60 | 0 |
| *Passer domesticus* | 50 | 50 | 0 | 0 | 0 |
| *Passerina ciris* | 50 | 50 | 0 | 0 | 0 |
| *Passerina cyanea* | 40 | 40 | 20 | 0 | 0 |
| *Passerina leclancherii* | 40 | 60 | 0 | 0 | 0 |
| *Pavo cristatus* | 100 | 0 | 0 | 0 | 0 |
| *Perdix perdix* | 100 | 0 | 0 | 0 | 0 |
| *Phaps chalcoptera* | 60 | 40 | 0 | 0 | 0 |
| *Phasianus colchicus* | 100 | 0 | 0 | 0 | 0 |
| *Ploceus philippinus* | 40 | 10 | 10 | 30 | 10 |
| *Poicephalus senegalus* | 20 | 20 | 40 | 20 | 0 |

**Appendix s2 (continued)**:

| Species | Ground | Understory | Midstory | Canopy | Aerial |
| --- | --- | --- | --- | --- | --- |
| *Probosciger aterrimus* | 10 | 10 | 40 | 40 | 0 |
| *Psittacula cyanocephala* | 33 | 33 | 33 | 0 | 0 |
| *Psittacula krameri* | 30 | 30 | 30 | 10 | 0 |
| *Psitticara acuticaudata* | 0 | 33 | 33 | 33 | 0 |
| *Psitticara erythrogenys* | 50 | 50 | 0 | 0 | 0 |
| *Psittirostra psittacea* | 0 | 20 | 30 | 50 | 0 |
| *Pycnonotus cafer* | 20 | 30 | 30 | 0 | 20 |
| *Pycnonotus jocosus* | 20 | 40 | 40 | 0 | 0 |
| *Rhipidura leucophrys* | 0 | 0 | 50 | 30 | 20 |
| *Rollulus rouloul* | 100 | 0 | 0 | 0 | 0 |
| *Serinus leucopygius* | 80 | 20 | 0 | 0 | 0 |
| *Serinus mozambicus* | 50 | 0 | 50 | 0 | 0 |
| *Sicalis flaveola* | 40 | 60 | 0 | 0 | 0 |
| *Spilopelia chinensis* | 100 | 0 | 0 | 0 | 0 |
| *Stagonopleura guttata* | 80 | 20 | 0 | 0 | 0 |
| *Streptopelia decaocto* | 80 | 10 | 10 | 0 | 0 |
| *Streptopelia roseogrisea* | 100 | 0 | 0 | 0 | 0 |
| *Syrmaticus reevesii* | 100 | 0 | 0 | 0 | 0 |
| *Syrmaticus soemmerringii* | 100 | 0 | 0 | 0 | 0 |
| *Taeniopygia guttata* | 0 | 0 | 20 | 80 | 0 |
| *Tiaris olivaceus* | 90 | 10 | 0 | 0 | 0 |
| *Tympanuchus cupido* | 100 | 0 | 0 | 0 | 0 |
| *Tyto alba* | 90 | 10 | 0 | 0 | 0 |
| *Uraeginthus angolensis* | 100 | 0 | 0 | 0 | 0 |
| *Uraeginthus cyanocephalus* | 100 | 0 | 0 | 0 | 0 |
| *Urocissa erythroryncha* | 30 | 20 | 50 | 0 | 0 |
| *Vidua chalybeata* | 0 | 0 | 90 | 10 | 0 |
| *Vidua macroura* | 100 | 0 | 0 | 0 | 0 |
| *Zenaida macroura* | 60 | 40 | 0 | 0 | 0 |
| *Zosterops japonicus* | 0 | 30 | 40 | 30 | 0 |

**Appendix s3**: Terrestrial bird introduction history data for Oʻahu, Hawaiʻi

Introduction history data for each bird reported as resident, extinct, or introduced but failed to establish by Pyle and Pyle (2017). Status was taken from Pyle and Pyle (2017) where N = native, X = extinct/extirpated native, I = established introduced, and IE = extirpated introduced species. The year of introduction and last year the species was recorded was taken from Pyle and Pyle (2017), Moulton and Pimm (1983), Moulton (1985), Simberloff and Boecklen (1991), Moulton (1993), and eBird (Sullivan et al. 2009). Whether or not the species was introduced via escaping (E), intentionally released (R), or both (B) is also shown. The number of years the bird was introduced is also reported with species where the data was not given having a number of years value = 1.

| Family | Scientific Name | Status | Individuals | Earliest Year | Last Year | Escape or Release | Number of Years |
| --- | --- | --- | --- | --- | --- | --- | --- |
| Accipitridae | *Buteogallus meridionalis* | IE | 1 | 1973 | 1973 | E | 1 |
| Alaudidae | *Alauda arvensis* | I | 58 | 1865 | 2018 | R | 3 |
| Apodidae | *Aerodramus bartschi* | I | 385 | 1962 | 2018 | R | 2 |
| Cacatuidae | *Cacatua alba* | IE | 4 | 1987 | 2018 | E | 1 |
| Cacatuidae | *Cacatua galerita* | IE | 11 | 1980 | 2018 | E | 4 |
| Cacatuidae | *Cacatua goffiniana* | IE | 30 | 1987 | 2018 | E | 1 |
| Cacatuidae | *Cacatua moluccensis* | IE | 11 | 1972 | 2018 | E | 38 |
| Cacatuidae | *Cacatua sulphurea* | IE | 2 | 1982 | 1983 | E | 1 |
| Cacatuidae | *Nymphicus hollandicus* | IE | 9 | 1979 | 2016 | E | 9 |
| Cacatuidae | *Probosciger aterrimus* | IE | 1 | 1983 | 1983 | E | 1 |
| Cardinalidae | *Cardinalis cardinalis* | I | 2 | 1929 | 2018 | B | 3 |
| Cardinalidae | *Passerina ciris* | IE | NA | 1937 | 1937 | R | 1 |
| Cardinalidae | *Passerina cyanea* | IE | NA | 1934 | 1936 | R | 3 |
| Cardinalidae | *Passerina leclancherii* | IE | 850 | 1941 | 1959 | R | 10 |
| Cettiidae | *Cettia diphone* | I | 138 | 1929 | 2018 | R | 12 |
| Columbidae | *Chalcophaps indica* | IE | 2 | 1924 | 1928 | R | 1 |
| Columbidae | *Columba livia* | I | NA | 1788 | 2018 | E | 2 |
| Columbidae | *Geopelia cuneata* | IE | 2 | 1928 | 1931 | R | 1 |
| Columbidae | *Geopelia humeralis* | IE | NA | 1920 | 1938 | R | 1 |
| Columbidae | *Geopelia placida* | IE | NA | 1922 | 1937 | R | 1 |
| Columbidae | *Geopelia striata* | I | NA | 1922 | 2018 | R | 1 |

**Appendix s3 (continued)**:

| Family | Scientific Name | Status | Individuals | Earliest Year | Last Year | Escape or Release | Number of Years |
| --- | --- | --- | --- | --- | --- | --- | --- |
| Columbidae | *Ocyphaps lophotes* | IE | 2 | 1922 | 1927 | R | 1 |
| Columbidae | *Phaps chalcoptera* | IE | 2 | 1922 | 1927 | R | 1 |
| Columbidae | *Spilopelia chinensis* | I | NA | 1855 | 2018 | B | 6 |
| Columbidae | *Streptopelia decaocto* | IE | 464 | 1924 | 1944 | R | 6 |
| Columbidae | *Streptopelia roseogrisea* | IE | 7 | 1974 | 1989 | E | 1 |
| Columbidae | *Zenaida macroura* | I | 2 | 2002 | 2018 | R | 1 |
| Corvidae | *Urocissa erythroryncha* | IE | 3 | 1967 | 1970 | E | 1 |
| Estrilidae | *Amandava amandava* | I | NA | 1900 | 2018 | E | 1 |
| Estrilidae | *Estrilda astrild* | I | 25 | 1973 | 2018 | E | 1 |
| Estrilidae | *Estrilda caerulescens* | IE | NA | 1965 | 2018 | E | 1 |
| Estrilidae | *Estrilda melpoda* | IE | 8 | 1965 | 2018 | E | 1 |
| Estrilidae | *Euodice cantans* | I | NA | 1984 | 2018 | E | 1 |
| Estrilidae | *Lagonosticta rubricata* | IE | 61 | 1965 | 1970 | E | 1 |
| Estrilidae | *Lonchura atricapilla* | I | 25 | 1959 | 2018 | E | 1 |
| Estrilidae | *Lonchura malacca* | IE | NA | 1967 | 1977 | E | 1 |
| Estrilidae | *Lonchura oryzivora* | I | 13 | 1964 | 2018 | E | 11 |
| Estrilidae | *Lonchura punctulata* | I | NA | 1866 | 2018 | R | 1 |
| Estrilidae | *Stagonopleura guttata* | IE | 26 | 1966 | 1966 | E | 1 |
| Estrilidae | *Taeniopygia guttata* | IE | NA | 2002 | 2002 | E | 1 |
| Estrilidae | *Uraeginthus angolensis* | IE | 12 | 1969 | 1977 | E | 1 |
| Estrilidae | *Uraeginthus cyanocephalus* | IE | 12 | 1969 | 1975 | E | NA |
| Fringillidae | *Akialoa ellisiana* | X | NA | NA | 1892 | NA | NA |
| Fringillidae | *Haemorhous mexicanus* | I | NA | 1859 | 2018 | R | 1 |
| Fringillidae | *Chlorodrepanis flavus* | N | NA | NA | 2018 | NA | NA |
| Fringillidae | *Drepanis coccinea* | N | NA | NA | 2013 | NA | NA |

**Appendix S3 (continued)**:

| Family | Scientific Name | Status | Individuals | Earliest Year | Last Year | Escape or Release | Number of Years |
| --- | --- | --- | --- | --- | --- | --- | --- |
| Fringillidae | *Hemignathus lucidus* | X | NA | NA | 1860 | NA | NA |
| Fringillidae | *Himatione sanguinea* | N | NA | NA | 2018 | NA | NA |
| Fringillidae | *Loxops wolstenholmei* | X | NA | NA | 1901 | NA | NA |
| Fringillidae | *Paroreomyza maculata* | X | NA | NA | 1968 | NA | NA |
| Fringillidae | *Psittirostra psittacea* | X | NA | NA | 1893 | NA | NA |
| Fringillidae | *Serinus leucopygius* | IE | 12 | 1965 | 1976 | E | 1 |
| Fringillidae | *Serinus mozambicus* | I | NA | 1964 | 2018 | B | 1 |
| Leiothrichidae | *Garrulax canorus* | I | NA | 1900 | 2018 | B | 2 |
| Leiothrichidae | *Garrulax chinensis* | IE | 5 | 1928 | 1928 | R | 1 |
| Leiothrichidae | *Garrulax leucolophus* | IE | 6 | 1969 | 1973 | E | 3 |
| Leiothrichidae | *Leiothrix lutea* | I | 230 | 1928 | 2018 | R | 1 |
| Mimidae | *Mimus polyglottos* | I | 84 | 1928 | 2018 | R | 6 |
| Mohoidae | *Moho apicalis* | X | NA | NA | 1837 | NA | NA |
| Monarchidae | *Chasiempis ibidis* | N | NA | NA | 2018 | NA | NA |
| Monarchidae | *Grallina cyanoleuca* | IE | 20 | 1924 | 1936 | R | 1 |
| Muscicapidae | *Copsychus malabaricus* | I | 84 | 1938 | 2018 | R | 3 |
| Muscicapidae | *Copsychus saularis* | IE | 16 | 1932 | 1976 | R | 9 |
| Muscicapidae | *Cyanoptila cyanomelana* | IE | NA | 1929 | 1958 | R | 11 |
| Muscicapidae | *Erithacus akahige* | IE | NA | 1929 | 1944 | R | 3 |
| Muscicapidae | *Erithacus komadori* | IE | NA | 1931 | 1936 | R | 1 |
| Muscicapidae | *Ficedula narcissina* | IE | NA | 1929 | 1929 | E | 1 |
| Nectariniidae | *Nectariniidae sp.* | IE | 28 | 1938 | 1939 | R | 2 |
| Numididae | *Numida meleagris* | IE | 25256 | 1900 | 2015 | R | 11 |
| Odontophoridae | *Oreortyx pictus* | IE | 28 | 1929 | 1931 | R | 2 |
| Oriolidae | *Oriolus chinensis* | IE | NA | 2000 | 2000 | E | 1 |

**Appendix S3 (continued)**:

| Family | Scientific Name | Status | Individuals | Earliest Year | Last Year | Escape or Release | Number of Years |
| --- | --- | --- | --- | --- | --- | --- | --- |
| Oriolidae | *Oriolus xanthornus* | IE | 2 | 2000 | 2000 | E | 1 |
| Paridae | *Parus varius* | IE | NA | 1929 | 1968 | E | 3 |
| Passeridae | *Passer domesticus* | I | 9 | 1871 | 2018 | R | 1 |
| Phasianidae | *Chrysolophus amherstiae* | IE | 49 | 1931 | 1932 | R | 1 |
| Phasianidae | *Chrysolophus pictus* | IE | NA | 1866 | 1866 | R | 2 |
| Phasianidae | *Colinus virginianus* | IE | 48 | 1962 | 1962 | R | 1 |
| Phasianidae | *Coturnix chinensis* | IE | NA | 1910 | 1944 | R | 1 |
| Phasianidae | *Coturnix japonica* | IE | 40 | 1866 | 1986 | R | 2 |
| Phasianidae | *Francolinus erckelii* | I | 100 | 1957 | 2018 | R | 10 |
| Phasianidae | *Francolinus francolinus* | I | NA | 1994 | 2018 | R | 1 |
| Phasianidae | *Francolinus pondicerianus* | I | NA | 1980 | 2018 | R | 1 |
| Phasianidae | *Gallus gallus* | I | 40 | 500 | 2018 | R | 125 |
| Phasianidae | *Lophura leucomelanos* | I | NA | 1987 | 2018 | E | 1 |
| Phasianidae | *Lophura nycthemera* | IE | NA | 1866 | 1995 | R | 3 |
| Phasianidae | *Meleagris gallopavo* | I | NA | 1788 | 2018 | B | 150 |
| Phasianidae | *Pavo cristatus* | I | NA | 1860 | 2018 | R | 1 |
| Phasianidae | *Perdix perdix* | IE | 1011 | 1929 | 1933 | R | 4 |
| Phasianidae | *Phasianus colchicus* | I | 10000 | 1866 | 2018 | R | 2 |
| Phasianidae | *Rollulus rouloul* | IE | 2 | 1924 | 1924 | R | 1 |
| Phasianidae | *Syrmaticus reevesii* | IE | 217 | 1959 | 1968 | R | 2 |
| Phasianidae | *Syrmaticus soemmerringii* | IE | NA | 1907 | 1914 | R | 2 |
| Phasianidae | *Tympanuchus cupido* | IE | 12 | 1895 | 1895 | R | 1 |
| Ploceidae | *Euplectis afer* | IE | 3 | 1965 | 1986 | E | 1 |
| Ploceidae | *Euplectis franciscanus* | IE | NA | 1965 | 2011 | E | 9 |
| Ploceidae | *Euplectis orix* | IE | NA | 1964 | 1994 | E | 1 |

**Appendix S3 (continued)**:

| Family | Scientific Name | Status | Individuals | Earliest Year | Last Year | Escape or Release | Number of Years |
| --- | --- | --- | --- | --- | --- | --- | --- |
| Ploceidae | *Ploceus philippinus* | IE | NA | 1965 | 1965 | E | 1 |
| Psittacidae | *Agapornis personatus* | IE | 2 | 1981 | 1984 | E | 1 |
| Psittacidae | *Agapornis roseicollis* | IE | NA | 1973 | 2016 | E | 29 |
| Psittacidae | *Amazona aestiva* | IE | 2 | 1988 | 2004 | E | 1 |
| Psittacidae | *Amazona albifrons* | IE | 5 | 1984 | 1985 | E | 2 |
| Psittacidae | *Amazona autumnalis* | IE | 2 | 1980 | 1987 | E | 2 |
| Psittacidae | *Amazona oratrix* | IE | 12 | 1969 | 2000 | E | 7 |
| Psittacidae | *Amazona viridigenalis* | I | 3 | 1969 | 2018 | E | 1 |
| Psittacidae | *Ara ararauna* | IE | 1 | 2005 | 2017 | E | 2 |
| Psittacidae | *Ara macao* | IE | 2 | 1933 | 2012 | E | 1 |
| Psittacidae | *Aratinga jandaya* | IE | 1 | 1974 | 1974 | E | 1 |
| Psittacidae | *Aratinga nenday* | IE | 7 | 1971 | 1987 | E | 1 |
| Psittacidae | *Brotogeris jugularis* | IE | NA | 1933 | 1933 | E | 1 |
| Psittacidae | *Cyanoramphus novaezelandiae* | IE | 1 | 1990 | 1990 | E | 1 |
| Psittacidae | *Eclectus roratus* | IE | 2 | 1972 | 2016 | E | 1 |
| Psittacidae | *Eupsittula canicularis* | IE | 2 | 1984 | 1985 | E | 2 |
| Psittacidae | *Melopsittacus undulatus* | IE | NA | 1933 | 2010 | E | 8 |
| Psittacidae | *Myiopsitta monachus* | IE | 11 | 1970 | 1977 | E | 1 |
| Psittacidae | *Poicephalus senegalus* | IE | 6 | 1984 | 1995 | E | 4 |
| Psittacidae | *Psittacula cyanocephala* | IE | 1 | 1992 | 1992 | E | 1 |
| Psittacidae | *Psitticara acuticaudata* | IE | 16 | 1986 | 2018 | E | 1 |
| Psittacidae | *Psitticara erythrogenys* | I | NA | 1987 | 2018 | E | 1 |
| Psittaculidae | *Psittacula krameri* | I | NA | 1930 | 2018 | E | 1 |
| Pycnonotidae | *Pycnonotus cafer* | I | NA | 1965 | 2018 | B | 1 |
| Pycnonotidae | *Pycnonotus jocosus* | I | NA | 1965 | 2018 | B | 1 |

**Appendix S3 (continued)**:

| Family | Scientific Name | Status | Individuals | Earliest Year | Last Year | Escape or Release | Number of Years |
| --- | --- | --- | --- | --- | --- | --- | --- |
| Rhipiduridae | *Rhipidura leucophrys* | IE | 14 | 1926 | 1937 | R | 2 |
| Strigidae | *Asio flammeus* | N | NA | NA | 2018 | NA | NA |
| Sturnidae | *Acridotheres tristis* | I | NA | 1866 | 2018 | R | 1 |
| Sturnidae | *Gracula religiosa* | IE | 25 | 1960 | 2009 | E | 6 |
| Sturnidae | *Gracupica nigricollis* | IE | 1 | 1969 | 1969 | E | 1 |
| Sturnidae | *Leucopsar rothschildi* | IE | 1 | 1975 | 1979 | E | 1 |
| Thraupidae | *Gubernatrix cristata* | IE | NA | 1965 | 1969 | E | 2 |
| Thraupidae | *Paroaria coronata* | I | NA | 1928 | 2018 | R | 4 |
| Thraupidae | *Paroaria dominicana* | IE | NA | 1931 | 1932 | R | 1 |
| Thraupidae | *Paroaria gularis* | IE | 2 | 1965 | 1965 | E | 1 |
| Thraupidae | *Sicalis flaveola* | I | NA | 1965 | 2018 | R | 1 |
| Thraupidae | *Tiaris olivaceus* | I | NA | 1974 | 2018 | E | 1 |
| Turdidae | *Myadestes woahensis* | X | NA | NA | 1825 | NA | NA |
| Tytonidae | *Tyto alba* | I | NA | 1958 | 2018 | E | 6 |
| Viduidae | *Vidua chalybeata* | IE | 1 | 1969 | 1969 | E | 1 |
| Viduidae | *Vidua macroura* | IE | 18 | 1962 | 1981 | E | 1 |
| Zosteropidae | *Zosterops japonicus* | I | NA | 1929 | 2018 | R | 2 |

**Appendix S4**: Average morphological measurements for each species
caught during mist netting

The average morphological measurements for the 17 species caught during mist netting from 2014 through 2017. The wing measurement was wing chord and the culmen measurement was taken from the joint of the culmen and cranium to the tip of the culmen. The nares to culmen tip measurement was taken from the distal edge of nares to the tip of the culmen. Bill width and depth were taken at the proximal edge of the nares. For some species mass was not taken from mist netting data and obtained from EltonTraits 1.0 (Wilman et al 2013).

| Species | Tail | Tarsus | Wing | Culmen | Nares to Culmen Tip | Bill Width | Bill Depth | Mass |
| --- | --- | --- | --- | --- | --- | --- | --- | --- |
| *Cardinalis cardinalis* | 98.55 | 23.82 | 90.47 | 18.16 | 13.11 | 10.27 | 13.32 | 42.64 |
| *Haemorhous mexicanus* | 58.33 | 18.34 | 75.90 | 11.49 | 8.41 | 7.29 | 8.12 | 21.40 |
| *Cettia diphone* | 55.61 | 22.69 | 56.03 | 12.20 | 7.05 | 4.15 | 3.59 | 13.31 |
| *Chlorodrepanis flavus* | 41.12 | 20.85 | 63.92 | 14.82 | 11.60 | 4.60 | 4.73 | 12.64 |
| *Copsychus malabaricus* | 116.75 | 25.41 | 87.78 | 17.03 | 10.92 | 5.36 | 5.38 | 29.70 |
| *Estrilda astrild* | 40.14 | 13.30 | 42.83 | 9.26 | 10.00 | 4.91 | 5.91 | 8.29 |
| *Garrulax canorus* | 89.30 | 36.40 | 86.70 | 21.17 | 13.77 | 6.10 | 7.23 | 62.78 |
| *Geopelia striata* | 95.46 | 19.73 | 96.83 | 15.44 | 8.65 | 4.14 | 4.04 | 56.60 |
| *Himatione sanguinea* | 48.95 | 22.53 | 71.19 | 16.66 | 12.40 | 5.22 | 4.56 | 15.17 |
| *Leiothrix lutea* | 54.92 | 23.74 | 66.59 | 13.15 | 7.68 | 5.08 | 5.02 | 21.10 |
| *Lonchura punctulata* | 37.98 | 14.06 | 52.54 | 11.85 | 8.58 | 6.81 | 8.12 | 13.60 |
| *Paroaria coronata* | 82.48 | 24.66 | 97.78 | 15.01 | 10.38 | 7.35 | 8.79 | 37.79 |
| *Pycnonotus cafer* | 89.13 | 23.43 | 94.76 | 19.29 | 10.42 | 6.37 | 5.91 | 37.20 |
| *Pycnonotus jocosus* | 77.25 | 20.37 | 79.13 | 16.28 | 8.89 | 5.52 | 5.06 | 26.30 |
| *Spilopelia chinensis* | 141.31 | 27.03 | 149.54 | 20.25 | 10.31 | 5.68 | 5.75 | 159.00 |
| *Tiaris olivaceus* | 41.40 | 15.50 | 49.40 | 9.70 | 6.35 | 4.50 | 6.10 | 8.50 |
| *Zosterops japonicus* | 41.19 | 17.54 | 58.04 | 13.12 | 7.70 | 4.27 | 3.66 | 10.80 |

**Appendix S5**: Ecological niche data taken from field observations of the focal species

The ecological data taken during the field observations of the five focal species at the four sites. For every data type except height, the probability of observing the behavior during the observations is given. The relative height (height observed divided by the average canopy height) is given for the max and min height. EKA = Ekahanui, MOA = Moanalua Valley, PAH = Pahole Natural Area Reserve, and WAI = Waimea Valley.

| Species | Site | Glean | Flycatch | Fruit | Hang | Hop | Interior | Periphery | Ground | Max Height | Min Height |
| --- | --- | --- | --- | --- | --- | --- | --- | --- | --- | --- | --- |
| *Zosterops   japonicus* | EKA | 0.838 | 0.027 | 0.054 | 0.206 | 0.529 | 0.333 | 0.833 | 0.019 | 0.617 | 0.536 |
|  | MOA | 0.870 | 0.043 | 0.130 | 0.348 | 0.522 | 0 | 1 | 0 | 0.479 | 0.376 |
|  | PAH | 0.760 | 0.080 | 0.040 | 0.106 | 0.541 | 0.375 | 0.813 | 0 | 0.759 | 0.653 |
|  | WAI | 0.833 | 0 | 0.111 | 0.100 | 0.400 | 0.455 | 0.636 | 0 | 0.547 | 0.434 |
|  | Total | 0.813 | 0.047 | 0.070 | 0.154 | 0.505 | 0.342 | 0.789 | 0.004 | 0.600 | 0.500 |
|  |  |  |  |  |  |  |  |  |  |  |  |
| *Leiothrix   lutea* | EKA | 1 | 0 | 0 | 0 | 0.773 | 0.667 | 1 | 0.065 | 0.426 | 0.263 |
|  | MOA | 0.864 | 0 | 0.227 | 0.018 | 0.636 | 1 | 0.143 | 0.122 | 0.283 | 0.182 |
|  | PAH | 0.750 | 0.125 | 0.250 | 0 | 0.659 | 0.667 | 0.333 | 0.022 | 0.512 | 0.380 |
|  | WAI | 0.333 | 0.222 | 0.667 | 0 | 0.652 | 0.667 | 0.333 | 0.172 | 0.418 | 0.260 |
|  | Total | 0.761 | 0.065 | 0.283 | 0.007 | 0.667 | 0.813 | 0.375 | 0.095 | 0.409 | 0.271 |
|  |  |  |  |  |  |  |  |  |  |  |  |
| *Pycnonotus   cafer* | EKA | 0 | 1 | 0 | 0 | 0.125 | 0 | 1 | 0 | 1.101 | 1.086 |
|  | MOA | 0.364 | 0.364 | 0.409 | 0 | 0.233 | 0.200 | 0.933 | 0 | 0.612 | 0.500 |
|  | PAH | 0.143 | 0 | 0 | 0 | 0.308 | 0.556 | 0.667 | 0 | 1.168 | 1.063 |
|  | WAI | 0.556 | 0.444 | 0.111 | 0.020 | 0 | 0.120 | 1 | 0 | 0.781 | 0.712 |
|  | Total | 0.341 | 0.366 | 0.244 | 0.009 | 0.140 | 0.220 | 0.920 | 0 | 0.915 | 0.840 |

**Appendix S5 (continued)**:

| Species | Site | Glean | Flycatch | Fruit | Hang | Hop | Interior | Periphery | Ground | Max Height | Min Height |
| --- | --- | --- | --- | --- | --- | --- | --- | --- | --- | --- | --- |
| *Pycnonotus   jocosus* | EKA | 0.333 | 0.833 | 0 | 0 | 0.091 | 0 | 1 | 0 | 0.534 | 0.441 |
|  | MOA | 0.531 | 0.344 | 0.313 | 0.088 | 0.455 | 0.111 | 1 | 0.065 | 0.489 | 0.378 |
|  | PAH | 0.250 | 0 | 0.750 | 0 | 0.154 | NA | NA | 0 | 0.731 | 0.686 |
|  | WAI | 0.167 | 0.333 | 0.500 | 0 | 0.138 | 0.200 | 0.900 | 0.030 | 0.672 | 0.554 |
|  | Total | 0.438 | 0.375 | 0.333 | 0.034 | 0.256 | 0.150 | 0.950 | 0.035 | 0.701 | 0.620 |
|  |  |  |  |  |  |  |  |  |  |  |  |
| *Copsychus   malabaricus* | EKA | NA | NA | NA | 0 | 0.250 | NA | NA | 0 | 0.227 | 0.227 |
|  | MOA | NA | NA | NA | 0 | 0.208 | 0 | 1 | 0.207 | 0.193 | 0.125 |
|  | PAH | NA | NA | NA | 0 | 0.143 | NA | NA | 0 | 0.270 | 0.199 |
|  | WAI | NA | NA | NA | 0 | 0 | NA | NA | 0.250 | 0.242 | 0.106 |
|  | Total | NA | NA | NA | 0 | 0.157 | 0 | 1 | 0.167 | 0.233 | 0.164 |
